# Host–virome associations in the weathering crust of a rapidly retreating temperate Alpine glacier

**DOI:** 10.1099/mgen.0.001524

**Published:** 2025-10-03

**Authors:** Gilda Varliero, Andreas Bauder, Beat Stierli, Weihong Qi, Beat Frey

**Affiliations:** 1 Rhizosphere Processes Group, Swiss Federal Institute for Forest, Snow and Landscape Research (WSL), Birmensdorf, Switzerland; 2 Laboratory of Hydraulics, Hydrology and Glaciology (VAW), ETH Zurich, Zurich, Switzerland; 3 Swiss Federal Institute for Forest, Snow and Landscape Research (WSL), Sion, Switzerland; 4 Functional Genomics Center Zurich, ETH Zurich and University of Zurich, Zurich, Switzerland; 5 Swiss Institute of Bioinformatics SIB, Geneva, Switzerland

**Keywords:** Alpine region, auxiliary metabolic gene (AMG), glacier, metagenomic island (MGI), metavirome, metaviromic island (MVI), weathering crust

## Abstract

Glaciers are retreating rapidly, altering ecosystem dynamics and increasing meltwater outflow into populated areas. Understanding microbial-virome interactions is crucial for predicting the consequences of this release. We sampled ice from four shallow pits in the weathering crust of the Rhonegletscher, Swiss Alps, and found a microbiome dominated by bacteria and microeukaryotes, alongside a metavirome infecting both groups. Viruses exhibited variable host specificity, with some targeting particular taxa and others showing a broader infectivity range. Variable genomic regions, including metagenomic and metaviromic islands, were enriched in genes related to replication, recombination, repair and transposable elements. Detected auxiliary metabolic genes were primarily involved in host coenzyme biosynthesis, uptake or utilization and in altering bacterial methylation patterns to evade detection. These findings underscore the major role of viruses in regulating microbial dynamics in glaciers and their potential downstream environmental impacts.

**Impact Statement**

As glaciers retreat at an accelerating pace, they release vast microbial and viral communities into downstream ecosystems, with unknown ecological consequences. Our study is the first one exploring the microbiome of the Rhonegletscher, and it reveals a virome deeply intertwined with bacterial and microeukaryotic hosts, influencing microbial dynamics through host-specific and broad-range infections. Viral genomes contain adaptive elements that drive genetic variability, including genes that modify host metabolism and evade immune detection. These findings highlight the crucial role of viruses in shaping glacier microbiomes and emphasize the need to understand their potential environmental impact as global warming alters glacial dynamics.

## Data Summary

All sequences can be found in the NCBI Sequence Read Archive under BioProject PRJNA1142417 (https://www.ncbi.nlm.nih.gov/bioproject/PRJNA1142417). The supplementary data consist of ‘Varliero_etal_supplementary_materials.docx’, reporting supplementary figures (Figs S1–S8, available in the online Supplementary Material) and results (Results S1 and S2), and ‘Varliero_etal_supplementary_tables.xlsx’, reporting all supplementary tables (Tables S1–S14).

## Introduction

Global glacier retreat is a critical environmental issue with far-reaching implications for ecosystems, water resources and human societies worldwide. The Central Alps, important freshwater sources for densely populated areas [1], will likely face significant socioeconomic impacts from glacier ice thawing. Switzerland harbours 1,463 glaciers, 90% of which are predicted to disappear by the end of this century [2]. In 2022 and 2023, Swiss glaciers lost over 10% of their overall volume [2]. The consequences of glacier retreat extend beyond hydrological changes, including the rapid dispersal of genetic material locked up in glaciers through river systems along watersheds. Understanding this process is crucial due to the possible release of human pathogens from these systems [3,4] and, more likely, the release of bacteria and viruses that may impact downstream systems through population dynamics and carbon and nutrient export [5,8]. Gaining deeper insight into the dynamics of the glacial biome is essential to clarify what is and will be released into the glacial downstream environments in the context of fast-retreating Alpine glaciers [9]. However, among Swiss glaciers, only the Dammagletscher, which has shown overall retreat since systematic monitoring started in 1921 and has experienced accelerated rates over the past two decades [2], has been the subject of microbiological studies [10,13].

Micro-organisms dominate glacial habitats and rely on nutrients and carbon deposited on glacier surfaces and released by photoautotrophic, chemoautotrophic and heterotrophic organisms [14]. On the glacial surface, glacial micro-organisms inhabit the weathering crust during the ablation season, an area of water-filled porous ice formed by solar radiation, which fosters a distinct microbial community [15,16]. Here, organisms play an active role in nutrient and carbon cycles particularly during the ablation season [17,19], when their metabolic activity is higher due to more favourable conditions. There are still many unknowns regarding glacial microbiomes [20,21], where most of these cold-adapted organisms are still uncharacterized [22]. In particular, viruses are a component of glacial communities that is dramatically understudied and yet important for glacial microbial dynamics.

Glacial viruses are diverse [23,25], with the majority being dsDNA bacteriophages, primarily belonging to the order Caudovirales [23]. However, other kinds of bacteriophages, such as ssDNA viruses [26], have also been found in glaciers. Additionally, viruses infecting protists, algae and fungi, also important components of the microbial community in glacial environments [14,27], have been identified [23,28]. Viruses have been found in both lytic and lysogenic phases. During the lytic phase, viruses enter the host cells and replicate using the host cell’s machinery, new viral particles are assembled and the host cell is lysed, releasing copies of the viruses back into the environment (i.e. virulent viruses). During the lysogenic phase, viral genomes are integrated into the host cell genome and are copied along with it, such that the viral genome is passed on to daughter cells (i.e. temperate viruses) [29,30]. The switch between the two lifestyles usually depends on changes in the environmental conditions, and while glacial viruses have mainly been found in the lytic phase [[25]], the lysogenic phase can be prevalent during less favourable environmental conditions (e.g. accumulation season) [31,32].

Virulent viruses exercise population control over microbial populations and, through the viral shunt, release carbon and nutrients into the environment, which is crucial in nutrient- and carbon-depleted environments such as glaciers [33,35]. High rates of infection and bacterial mortality caused by viruses can occur in glacial water systems [36,37]. Estimates of viral-induced mortality in microbial populations in the photic zone vary between 342 and 2606% in cryoconite hole sediments [34], and viral control can account for 35.5% of bacterial production in supraglacial meltwater [35]. In the photic zone of glaciers, viruses have also been observed to adopt a strategy of prolonged survival, over reproduction, to increase the probability of encountering cell hosts; this suggests that glacial viruses can be exported to downstream systems, where they remain viable and can infect organisms [5,35].

Metaviromic islands (MVIs) and metagenomic islands (MGIs) are metagenomic regions where alignment algorithms under-recruit reads, indicating high variability [38]. Viral populations can be similar among distant cryoconite holes, and MVIs are essential for viral diversity and elude host cell defences, enabling continued infection [38]. Likewise, micro-organisms can also exhibit variable genomic regions (i.e. MGIs) to promote different responses, such as adaptation to changing external conditions, escape from viral infection through fast mutations in viral-targeted regions or increase their pathogenicity [39]. MGIs can be acquired via horizontal gene transfer [39] and can also develop and be maintained in a population due to viral pressure [40]. Genomic links between most viruses and prokaryotes in various glacial-related environments of the Tibetan Plateau have been observed [25]. During this exchange of genes, metabolic genes can also be transferred. Viral genomes can contain auxiliary metabolic genes (AMGs) that help the virus survive once inside the host cell but can also benefit the host cell [41]. These AMGs have been found in the glacial environment, from supraglacial settings [25] to ancient ice [42].

 Here, we explored host–virus interactions in a glacier by investigating the microbial and viral communities of four shallow ice samples taken from the weathering crust of the Rhonegletscher in the Swiss Alps. Given the limited number of samples, our study aimed to provide an initial insight into the glacial microbiome and virome of the Rhonegletscher. Specifically, we investigated virus–host associations within the Rhonegletscher communities and examined the levels of viral specificity to host cells. To achieve this, we analysed genomic signatures related to virus–host infectivity trends, including MGIs, MVIs and AMGs.

## Methods

### Sample collection

Glacier ice samples were collected from the Rhonegletscher (46° 35.18′ N 8° 23.14′ E), which is located in the Central Alps in Switzerland at 2,300 m a.s.l., in the Grimsel granodiorite rocks of the Aar Massif [43] (Fig. S1). After a stable period between the 1960s and 1980s, the Rhonegletscher started to respond in the 1990s to the effects of global warming with a retreat behind a rock barrier where a proglacial lake has formed since 2000. During the peak of the Little Ice Age, the Rhonegletscher extended ~2.5 km downstream of its current terminus, reaching nearly to the hamlet of Gletsch [2].

Ice sampling took place in August 2022, after melting of the winter snow cover at the glacier surface. Three sampling locations were randomly identified in the glacial ablation zone, each ~50 m apart (Fig. S1). The first 10 cm of the sampled glacial ice exposed to the atmosphere was removed and discarded. Ice blocks down to a depth of 80 cm were then collected from the three locations and divided into surface (10–30 cm) and deeper ice samples (40–80 cm). Sampling of glacial ice was performed using ice axes whose surfaces had been sterilized. A total of six ice samples were collected, i.e. three surface samples and three deeper samples. Ice samples were transferred into sterile polypropylene bags (autoclavable decontamination bags, 72 l, 700×1,100 mm; Faust Laborbedarf AG, Schaffhausen, Switzerland) and were subsequently kept frozen during transport and stored at −80 °C [1] until DNA and RNA extraction. Subsequently, the ice was melted at 4 °C overnight on a sterile bench. Approximately 6–8 l of melted ice was collected per sample and filtered using gamma-sterilized Steritop-GP Express Plus filtration systems (Steritop-GP 500 ml, polyethersulfone, 0.22 µm pore size; Merck Millipore, Darmstadt, Germany). Cell-containing membranes were stored at −80 °C until isolation.

Anion concentrations in the melted glacial ice were measured using ion chromatography (ICS 3000, Dionex), pH with a pH electrode and stable isotopes with an isotope ratio mass spectrometer (Thermo Fisher Scientific, Waltham, MA, USA). Results of the ice chemistry analyses are presented in Results S1.

### Isolation and sequencing

Total genomic DNA and RNA from the glacier ice samples were extracted from the membrane filters. RNA was isolated from the filters using the RNeasy PowerSoil Total RNA kit (Qiagen, Hilden, Germany), and DNA was eluted from the same samples using the RNeasy DNA Elution kit (Qiagen), according to the manufacturer’s instructions. The concentrations of extracted DNA and RNA were quantified with a Qubit 3.0 fluorometer using a Qubit dsDNA HS Assay kit and a Qubit RNA HS Assay kit, respectively (Thermo Fisher Scientific). DNAse treatment of the isolated RNA was performed using ThermoFisher DNAfree kit (AM1906) according to the manufacturer’s instructions. To check that no contamination was introduced into the glacier samples during sample handling and processing, frozen sterile water (instead of glacial ice) was subjected to the above procedures. The concentration of the obtained DNA and RNA was measured and was below the detection limit of the Qubit dsDNA HS Assay kit and a Qubit RNA HS Assay kit (Thermo Fisher Scientific). The obtained DNA was also checked with a PCR using the 16S rRNA gene primers 341F (CCTAYGGGDBGCWSCAG) and 806R (GGACTACNVGGGTHTCTAAT) [44]. However, no visible amplification bands were obtained. Libraries were prepared for only four samples because two samples of deeper ice and the negative controls had DNA and RNA concentrations that were too low for whole shotgun metagenomic and metatranscriptomic library preparation. Ice samples 1, 2 and 3 were from a depth of 10–30 cm, and ice sample 4 was from a depth of 40–80 cm. Taxonomic and functional patterns across the two different sampling depths were not explored, as this was beyond the scope of the study and due to the lack of replicates. The first 10 cm was discarded to avoid confounding effects of atmospheric deposition (e.g. dry and wet deposition) [45].

Total RNA was rRNA-depleted using Ribo-Zero rRNA Removal Kit (Illumina, Inc., San Diego, CA, USA). DNA and RNA libraries were then prepared using the TruSeq DNA library preparation and TruSeq stranded RNA library preparation kits (Illumina). DNA and RNA were sequenced on an Illumina NovaSeq 6000 sequencer (2×100 bp). More than 81 million paired reads were obtained per DNA sequencing sample (i.e. DNA1, DNA2, DNA3 and DNA4), and more than 13 million paired reads were obtained per RNA sequencing sample (i.e. RNA1, RNA2, RNA3 and RNA4) (Table S1A).

### Bioinformatics

A schematic view of the bioinformatic pipeline is shown in Fig. S2. All DNA and RNA sequencing samples were quality-checked using Trimmomatic v0.39 [46]. DNA reads from all samples were assembled using MEGAHIT v1.2.9 [47], whereas RNA reads were assembled using Trinity v2.4.0 [48]. Assembly details are provided in Table S1B, C. Both DNA and RNA reads were then mapped back to the DNA assembly using BWA v0.7.17 [49], and read counts associated with each contig were obtained using SAMtools v1.18 with the subcommand ‘idxstats’ [50]. DNA and RNA abundances are reported as tags per million (TPM), where the counts of reads that mapped to the contigs were normalized by the length of the contigs and sequencing depth. Taxonomic annotation of all contigs was performed using Kaiju v1.9.2 [51]. The reference database was constructed from the NCBI non-redundant protein database including eukaryotes (accessed on 24 May 2023) using the command ‘kaiju-makedb -s nr_euk’.

#### Metagenome-assembled genomes

Metagenome-assembled genomes (MAGs) were obtained using three binners, CONCOCT v1.1.0 [52], MetaBAT2 v2.15 [53] and MaxBin v2.2.7 [54], considering only contigs longer than 1,500 bases. The obtained bins were then combined using DAS Tool v1.1.6 [55], and contamination was removed using MAGpurify v2.1.2 [56]. The completeness and contamination levels of each bin were checked using CheckM2 v1.0.1 [57], and the taxonomy was checked using GTDB-Tk v2.1.1 [58]. Only medium- and high-quality MAGs were investigated further; high-quality MAGs were defined as those with completeness >90% and contamination <5%, whereas medium-quality MAGs were all not defined as high quality and with completeness ≥50% and contamination <10% [59,60]. In total, 93 MAGs fulfilled these criteria and were named ‘Ice’ followed by sequential numbers from 1 to 93, with the smallest number assigned to the MAG with the highest completeness and the largest number to the MAG with the lowest. MAGs were annotated using DRAM v1.4 [61]. Predicted proteins were annotated to the eggNOG database v5.0.2 using eggNOG-mapper v2.1.12 [62]. Both DNA and RNA reads were subsequently mapped back to the MAGs using BWA v0.7.17 [49]. MAG coverages were calculated using BEDtools v2.31.1 with the subcommand ‘genomecov’ [63].

A phylogenetic tree containing all MAGs was constructed using PhyloPhlAn v3.0.68 [64]. Genomes for the tree construction were downloaded from the NCBI genome repository (date 26 March 2024) [65] following these rules: first, one genome per family was downloaded for all the phyla ascribed to the MAGs; then, one genome per genus was downloaded for all the families ascribed to the MAGs; further, some NCBI genomes identified by the software GTDB-Tk during the taxonomic annotation were also downloaded. A total of 919 NCBI downloaded genomes plus 93 MAGs were used to construct the phylogenetic tree. PhyloPhlAn was run using --min_num_markers 60 using the tree-of-life approach. MAG placement in the tree was also used to verify the taxonomy assigned by the GTDB-Tk software.

#### Viral operational taxonomic units

Viral contigs were retrieved using VirSorter2 v2.2.4 [66], VIBRANT v1.2.1 [67], Seeker v1.0 [68] and DeepVirFinder v1.0 [69], using default parameters and screening contigs retrieved by both metagenomic and metatranscriptomic assemblies (contig length ≥5,000 bases). The results were then filtered using the following thresholds: score ≥0.7 for VirSorter2, score ≥0.9 and *P*≤0.05 for DeepVirFinder and score ≥0.9 for Seeker. All putative viral contigs were then combined. Nonredundant viral operational taxonomic units (vOTUs) were obtained using the ‘cd-hit-est’ command from CD-HIT v4.8.1 [70,71], clustering them at a 95% identity threshold with at least 85% alignment coverage of the shorter contig.

CheckV v1.0.1 using database v1.5 [72] and VirSorter2 v2.2.4 [66] were run to assess vOTU quality. Using the parameters obtained from these tools, vOTUs were retained if they met at least one of the following criteria: contained at least one viral gene, contained no viral gene and no host gene or contained no viral gene but had a VirSorter2 score ≥0.95 or more than two hallmark viral genes (https://www.protocols.io/view/viral-sequence-identification-sop-with-virsorter2-5qpvoyqebg4o/). Lastly, only vOTUs equal or longer than 5,000 bases were kept. DRAM-v v1.4 [61] was run to predict coding regions and proteins which were then annotated to the eggNOG database v5.0.2 using eggNOG-mapper v2.1.12 [62].

Taxonomy was assigned using blastp v2.2.31+ [aligning the viral proteins against the RefSeq viral protein database (updated 15 January 2024) [65,73] and retaining alignments with bit-score ≥50], using blastn v2.2.31 (aligning the viral genes against the IMG/VR v4 database [73,74] and retaining alignments with percent identity >90%), using MMseqs2 v15.6 [75] (retaining alignments with bit-score ≥50) and using genoMAD v1.8.0 [76] (retaining alignments with ‘virus_score’ >0.7). Taxonomy was assigned to each based on the results of these four taxonomic classification methods. A taxonomic level was assigned to a vOTU only if all classification methods that could assign a taxonomy agreed; otherwise, it was labelled as ‘unclassified’. To assign DNA and RNA abundances to vOTUs, the reads were mapped back to the vOTUs using BWA v0.7.17 [49], and the number of reads was then associated with each vOTU using SAMtools v1.18 with the subcommand ‘idxstats’ [50].

#### Virus–host relationships

All virus–host relationship analyses were done using the MAGs rather than the entire assembly because the assembly could have included contigs that were later polished and identified as vOTUs. Further, MAGs are more polished and allow a better comparison of viral and microbial coding sequences (CDSs) compared with the less well-defined assembly contigs. Virus–host relationships were investigated using iPHOP v1.3.3 [77] with the default iPHOP database, with the addition of the 93 medium- and high-quality MAGs from this study. BACPHLIP v1 was used to identify the virus lifestyle (viral vs temperate viruses) [78]; only annotations with a score ≥0.80 were considered valid.

To investigate the relationships between viruses and eukaryotes, matching coding genes and tRNA sequences between vOTUs and eukaryotic genes were identified, because no MAGs were obtained from the eukaryotic components. tRNA sequences were obtained from vOTUs and eukaryotic contigs (retrieved from the Kaiju output) using ARAGORN v1.2.41 [79]. Genes predicted using DRAM-v were used for viruses (as described above), and MetaGeneMark v3.38 [80,81] was run for eukaryotic contigs. blastn v2.2.31+ [73] (-perc_identity 90 -qcov_hsp_perc 90) was then used to determine the correspondences between the two datasets. Only matches with the two main viral classes of our dataset that infect eukaryotes (i.e. Megaviricetes and Revtraviricetes) were analysed.

In order to retrieve the vOTU CDSs that were also present in the MAGs, blastp v2.2.31+ (--id 80 --query-cover 80) was used to align the MAG proteins against the viral proteins [73]. To define which processes were ascribed to the vOTU proteins found in the MAGs, the eggNOG classification [82] was used. AMGs were identified using DRAM-v v1.4 [61] and VIBRANT v1.2.1 [67]. The AMGs detected by both tools were combined, and only those not located at the vOTU ends were retained. The AMGs were then classified using the eggNOG classification, to ensure consistency with the other results reported in this article. Some predicted proteins were assigned to more than one eggNOG category; in such cases, one hit was assigned to each of the eggNOG categories ascribed to that specific protein (the same approach was used for the eggNOG-classified predicted proteins associated with MGIs and MVIs, see section below).

#### MGIs and MVIs

To find MGIs and MVIs, the protocol proposed by Bellas *et al*. [38] was partially followed [38]. First, all DNA reads were mapped to the MAGs (MGIs) or vOTUs (MVIs) using BWA v0.7.17 [49], and then, the alignments containing two or more mismatches were removed. Coverage of reads mapped to each MAG and vOTU was calculated using BEDtools v2.31.1 with the ‘genomecov’ subcommand. The coverage data were then screened for drops in coverage across the MAGs and vOTUs. An MGI (or MVI) was defined as a stretch of ≥100 bases where the coverage dropped ≥25% compared with the genome mean coverage, for genomes with a mean coverage ≥5×. If the mean genome coverage was between 2× and 5×, an MGI (or MVI) was defined as a continuous stretch of ≥200 bases with zero coverage [38]. Genes ascribed to MGIs and MVIs were defined as any gene fully or partially contained in these regions. These genes were annotated using the eggNOG classification [82], as described above.

### Data representation and statistical analyses

All data plots were generated in the R environment v4.2.2 [83]. The R packages ggplot2 v3.5.0 [84], ggpubr v0.6.0 [85], ComplexHeatmap v2.14.0 [86] and svglite v2.1.2 [87] were used to create the plots; readr v2.1.5 [88], tidyr v1.3.1 [89] and dplyr v1.1.4 [90] were used to format and import the data. The MAG phylogenetic tree was first plotted in R using the packages ape v5.7.1 [91] and ggtree v3.6.2 [92] and then explored using Dendroscope v3 [93]. The virus–bacteria Sankey plot was generated using ggplot2 v3.5.0 [84] and ggsankey v0.0.9 [94]. The phylogenetic tree and the other plots were finalized using Inkscape v1.2 (https://inkscape.org). Alpha diversity indices were calculated using the R package vegan v2.6-4 [95].

## Results

### Ice microbial taxonomy

**Fig. 1.**
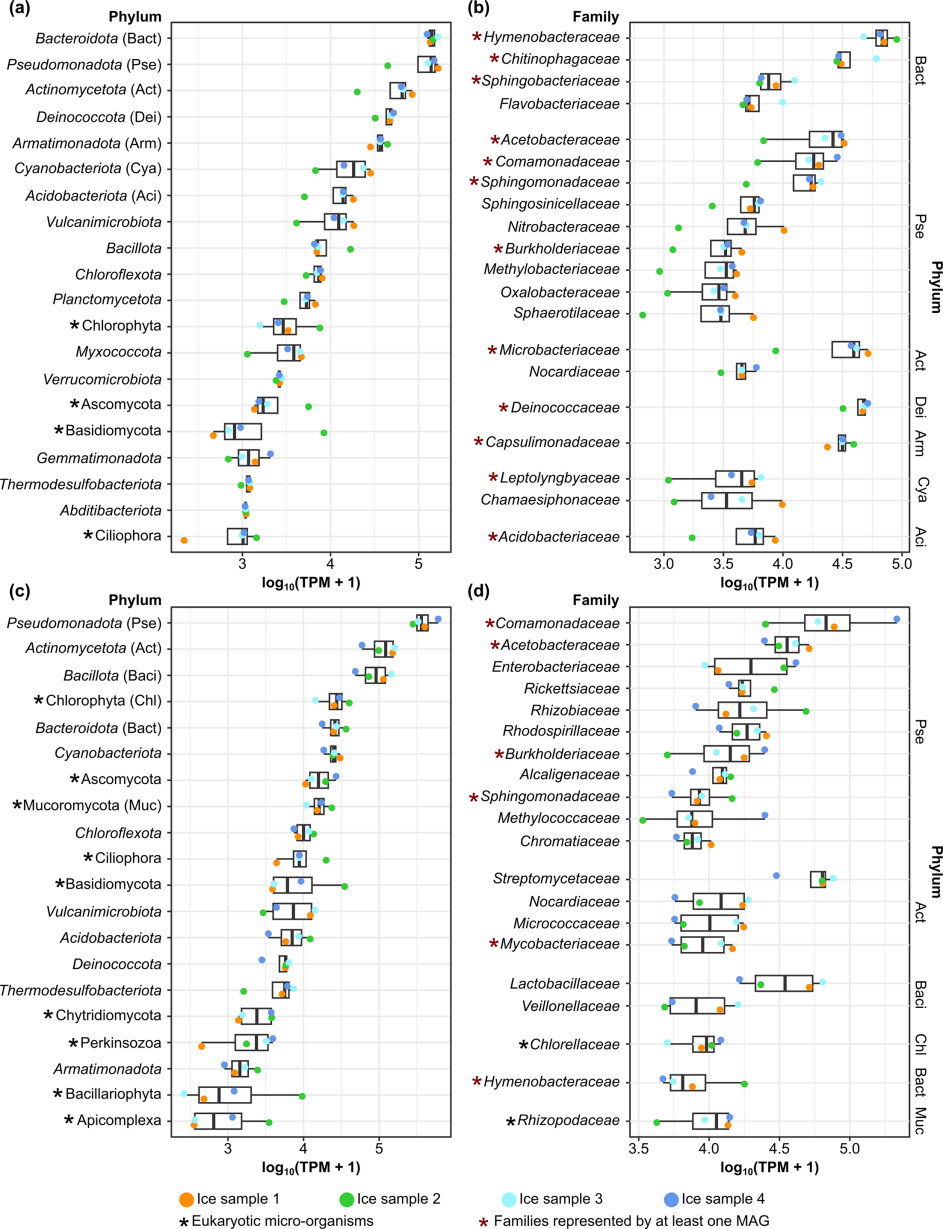
The 20 most abundant phyla and families in the DNA (**a, b**) and RNA (**c, d**) datasets. Phyla are ordered by decreasing mean TPM. Families are grouped by their respective phyla, with the phyla sorted by decreasing mean TPM. Within each phylum, families are listed in order of decreasing mean TPM. TPM values were transformed and shown as log_10_(TPM+1). The median and quartiles for each taxon are displayed in the box plots (*n*=4).

The largest portion of DNA reads was aligned to the phylum *Bacteroidota*, followed by *Pseudomonadota*, *Actinomycetota*, *Deinococcota*, *Armatimonadota* and *Cyanobacteriota* (Fig. 1a). The prokaryotic families strongly represented in the phylum *Bacteroidota* were *Hymenobacteraceae*, *Chitinophagaceae*, *Sphingobacteriaceae* and *Flavobacteriaceae*. *Pseudomonadota* was represented mainly by *Acetobacteraceae*, *Comamonadaceae* and *Sphingomonadaceae*; *Actinomycetota* by *Microbacteraceae* and *Nocardiaceae*; *Deinococcota* by *Deinococcaceae*; *Armatimonadota* by *Capsulimonadaceae*; and *Cyanobacteriota* by *Leptolyngbyaceae* and *Chamaesiphonaceae* (Fig. 1b). The RNA dataset showed different microbial profiles from the DNA dataset (Fig. 1). The most active prokaryotic phylum was *Pseudomonadota*, followed by *Actinomycetota*, *Bacillota*, *Bacteroidota* and *Cyanobacteriota* (Fig. 1c). Similarly, the prokaryotic families showed patterns different from those based on DNA, with *Comamonadaceae*, *Acetobacteraceae*, *Enterobacteriaceae*, *Rickettsiaceae* and *Rhizobiaceae* (*Pseudomonadota*), *Streptomycetaceae* (*Actinomycetota*) and *Lactobacillaceae* (*Bacillota*) emerging as the most active (Fig. 1d).

Four eukaryotic phyla were among the 20 most abundant phyla when DNA profiles were considered, whereas 8 occurred in the RNA mapping profiles (Fig. 1). Namely, Chlorophyta, Basidiomycota, Ascomycota and Ciliophora were present in both profiles, and Mucoromycota, Bacillariophyta, Perkinsozoa and Apicomplexa were present only in the RNA profiles (Fig. 1). At the family level, *Chlorellaceae* (Chlorophyta) and *Rhizopodaceae* (Mucoromycota) were among the 20 most abundant in the RNA dataset (Fig. 1d).

### Metagenome-assembled genomes

**Fig. 2.**
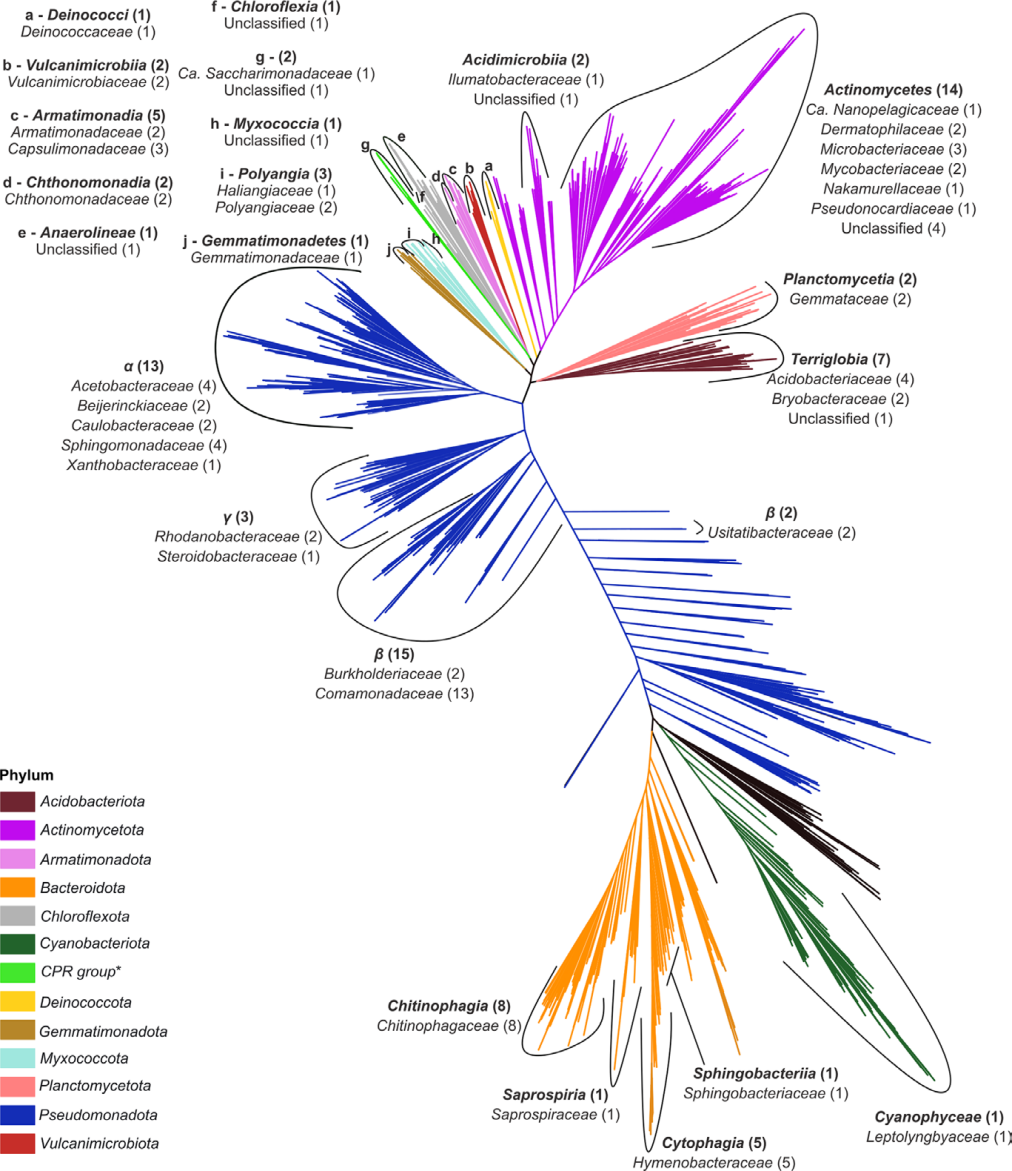
MAG placement in the phylogenetic tree. Different colours indicate the phylum-level classifications of the clusters. MAG taxonomic annotation at the class level is shown in bold, with the number of associated MAGs indicated in brackets. The taxonomic classification at the family level and the number of MAGs ascribed to each family (in brackets) is reported below the corresponding class. *Not phylum level.

We reconstructed a total of 43 medium-quality and 50 high-quality MAGs (Table S2). The MAGs spanned 12 phyla and had 2 representatives in the candidate phyla radiation (CPR) group (Fig. 2, Table S2). Most of the MAGs belonged to *Pseudomonadota* (33 MAGs assigned to 3 classes), *Bacteroidota* (16 MAGs assigned to 4 classes), *Actinomycetota* (16 MAGs assigned to 2 classes), *Armatimonadota* (7 MAGs assigned to 2 classes) and *Acidobacteriota* (7 MAGs assigned to 1 class). The MAG with the highest base coverage for both DNA and RNA data was Ice41, of the family *Vulcanimicrobiaceae* (phylum *Vulcanimicrobiota*; Fig. S3). The other MAGs with high coverage of DNA reads (i.e. coverage ≥100× in at least one sample) were Ice54 (*Microbacteriaceae*), Ice44 and Ice49 (*Capsulimonadaceae*), Ice80 and Ice91 (*Chitinophagaceae*), Ice52 and Ice68 (*Hymenobacteraceae*), Ice18 (*Sphingobacteriaceae*), Ice1 (*Leptolyngbyaceae*), Ice65 (*Acetobacteraceae*) and Ice29 (*Sphingomonadaceae*; Fig. S3A). Apart from Ice41, another MAG that showed high coverage of RNA reads was Ice48, from *Candidatus Saccharimonadaceae* (Fig. S3B). The reconstructed MAGs represented a major portion of the microbial community, as 46.5–69.3% of the DNA reads and 56.4–86.3% of RNA reads were associated with MAGs (Table S3). Thirteen of the families represented by at least 1 MAG were also among the 20 most abundant families in the DNA and RNA datasets, respectively (Fig. 1). Predicted MAG metabolic pathways for energy production and adaptations to the glacial environment are reported in Results S2.

### Ice viral taxonomy

**Fig. 3.**
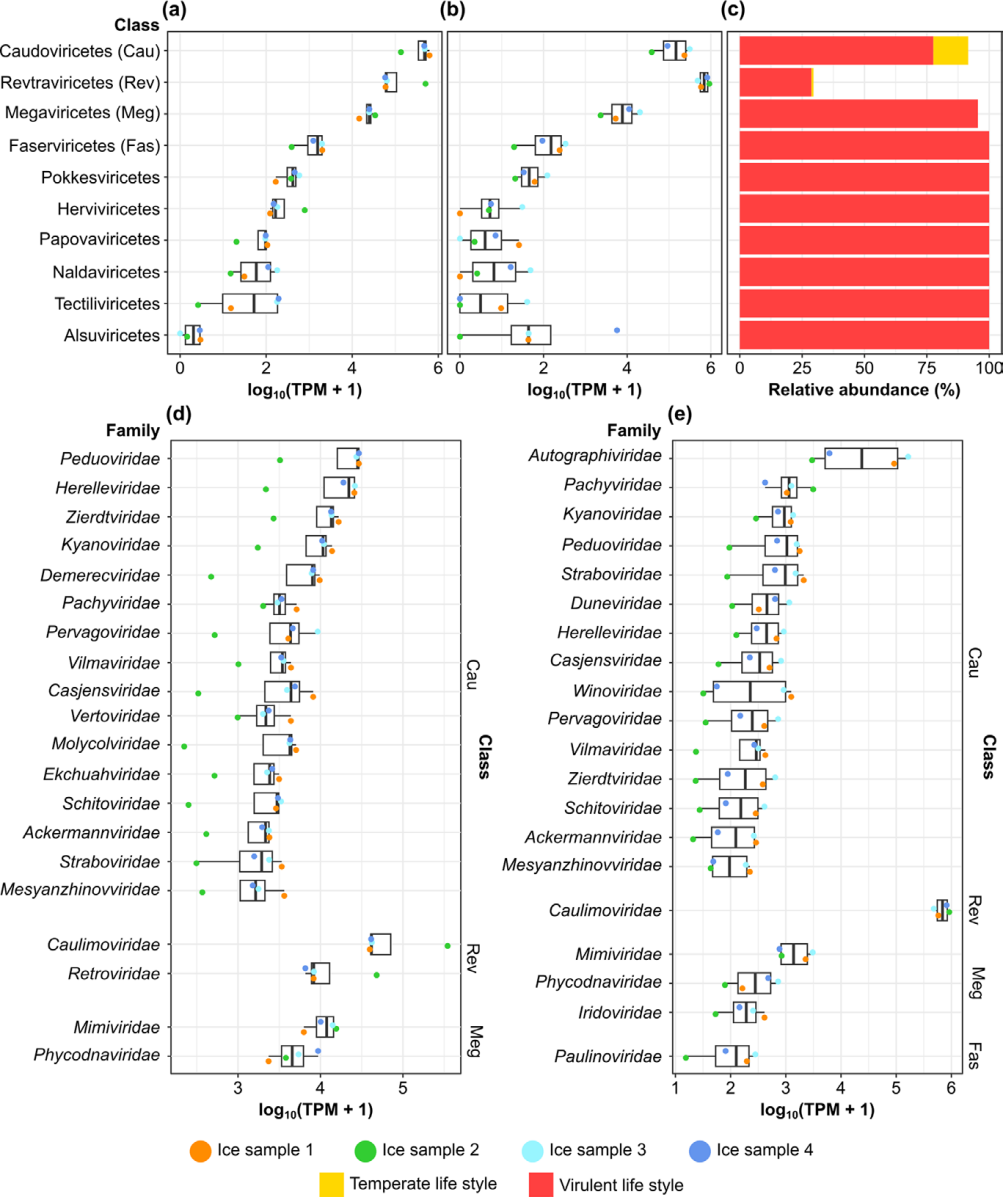
Virus relative abundance for the DNA (**a**) and RNA (**b**) datasets at the class level, along with the percentages of virulent and temperate viruses associated with each class (**c**). Classes are ordered by decreasing mean TPM in the DNA dataset. The 20 most abundant families for the DNA (**d**) and RNA (**e**) datasets. The classes associated with each family are indicated with abbreviations. TPM values were transformed and displayed as log_10_(TPM+1). The median and quartiles for each taxon are displayed in the box plots in (a), (b), (d) and (e) (*n*=4).

In our analysis, we reconstructed 2,565 vOTUs (Fig. S4, Tables S4 and S5). Of these, 46.7%, 46.2% and 17.2% were classified at the phylum, class and family levels, respectively. Most vOTUs were assigned to the class Caudoviricetes (892), followed by Megaviricetes (155) and Revtraviricetes (115) (Table S5). Based on the DNA dataset, the most abundant classes were Caudoviricetes (phylum Uriviricota), Revtraviricetes (Arterviricota) and Megaviricetes (Nucleocytoviricota); similar patterns emerged from the RNA dataset, although Revtraviricetes was more abundant than Caudoviricetes (Fig. 3a, b). All viral classes in our dataset showed a preponderance of virulent viruses, although the class Caudoviricetes had a higher proportion of temperate viruses (Fig. 3c). Overall, there were also more vOTUs associated with virulent viruses (2,184) than with temperate viruses (152); the remaining vOTUs could not be associated with either group.

Following the class-level taxonomic trends, the most abundant families in terms of DNA abundance belonged to Caudoviricetes (*Peduoviridae*, *Herelleviridae*, *Zierdtviridae*, *Kyanoviridae* and *Demerecviridae*; Fig. 3d), Revtraviricetes (*Caulimoviridae* and *Retroviridae*) and Megaviricetes (*Mimiviridae*). Based on the RNA dataset, the active viral families were spread across *Caulimoviridae* (Revtraviricetes), *Mimiviridae* (Megaviricetes) and *Autographiviridae*, *Pachyviridae*, *Kyanoviridae*, *Peduoviridae* and *Straboviridae* (Caudoviricetes) (Fig. 3e). Among the above-mentioned most abundant families, *Peduoviridae*, *Retroviridae*, *Kyanoviridae*, *Pachyviridae*, *Duneviridae* and *Autographiviridae* exhibited a temperate lifestyle, according to their genomic signatures (Figs S5 and S6).

### Viral relationships with bacterial MAGs

Of all the analysed vOTUs, 218 (8.5%) were inferred as potentially infecting bacterial and archaeal genomes. The microbial families that were most targeted by the viruses were *Hymenobacteraceae* (68), *Mycobacteriaceae* (20), *Comamonadaceae* (15), *Chitinophagaceae* (12) and *Microbacteriaceae* (10) (Table S6).

**Fig. 4.**
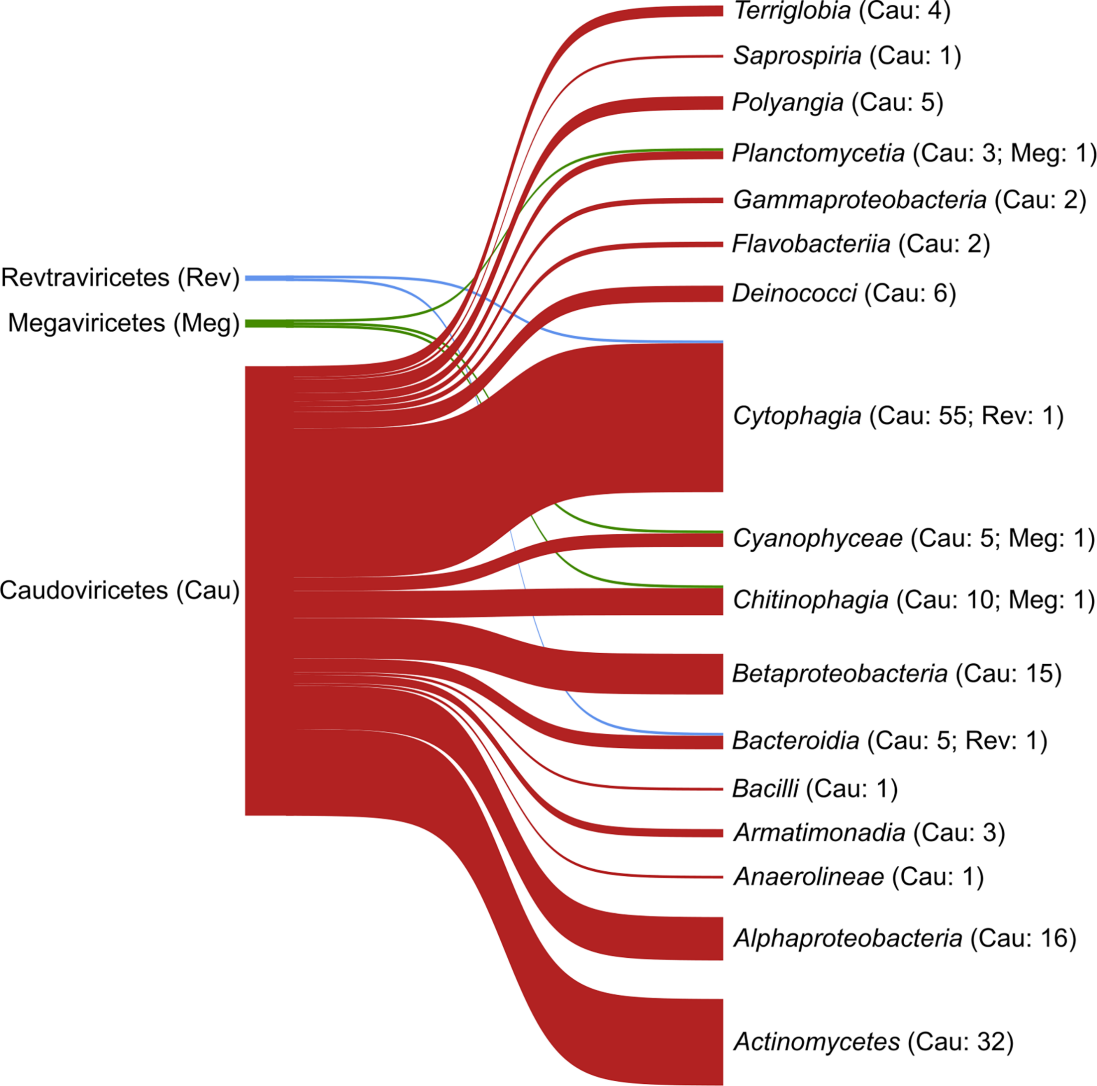
Number of vOTUs classified at the class level that were associated with bacterial classes. Viral classes associated with only one bacterial taxon are not included in the plot.

The three most abundant viral classes in both the DNA and RNA profiles (Fig. 4) were inferred as potentially infectious towards several microbial classes. The viral class Caudoviricetes showed 55 vOTUs infectious towards *Cytophagia*, 32 towards *Actinomycetes*, 16 towards *Alphaproteobacteria*, 10 towards *Chitinophagia* and 6 towards *Cyanophyceae*. Caudoviricetes potentially infected 17 microbial classes (14 excluding single-hit associations; Table S6), with 12 infected solely by this viral class. The viral class Megaviricetes was linked to three microbial classes (i.e. *Planctomycetia*, *Cyanophyceae* and *Chitinophagia*) and the viral class Revtraviricetes to two microbial classes (i.e. *Cytophagia* and *Bacteroidia*) (Fig. 4). Virus–bacteria interactions at the family level are reported in Fig. S7.

**Fig. 5.**
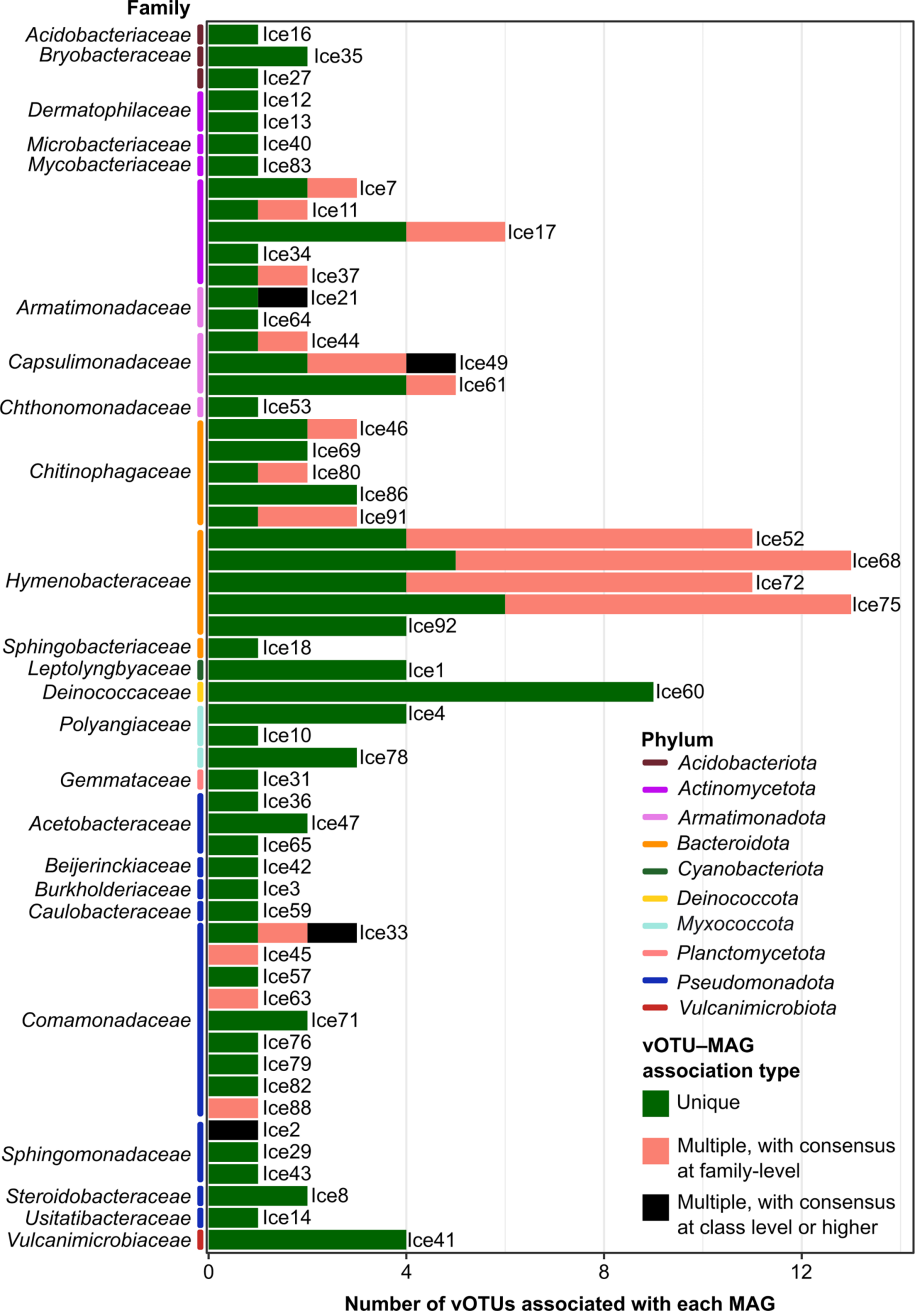
Associations between vOTUs and MAGs. vOTU-MAG association types are defined as follows: ‘Unique’ when a vOTU targets only one MAG; ‘Multiple, with consensus at the family level’ when a vOTU targets different MAGs assigned to the same family; and ‘Multiple, with consensus at class level or higher’ when a vOTU targets MAGs assigned to different classes or higher taxonomic levels. MAG names (Ice1 to Ice93) are reported to the right of the bar plots.

When MAGs were considered, 56 of them matched at least 1 vOTU. The 37 MAGs that did not match any of the vOTUs belonged to 11 phyla. Among them, only MAGs belonging to *Chloroflexota*, the CPR group and *Gemmatimonadota* had no matches. The MAGs associated with the largest number of vOTUs belonged to the families *Hymenobacteraceae* (Ice52, Ice68, Ice72 and Ice75), *Deinococcaceae* (Ice60) and *Capsulimonadaceae* (Ice49 and Ice61). Most MAGs were uniquely matched by vOTUs, except for Ice45, Ice63, Ice88 and Ice2. 67.8% of the vOTUs uniquely matched one MAG, 29.7% matched different MAGs within the same bacterial family (e.g. *Capsulimonadaceae*, *Chitinophagaceae*, *Hymenobacteraceae* and *Comamonadaceae*), and 2.5% matched several MAGs from different families (Fig. 5). Of the 56 MAGs associated with at least one vOTU, 47 were linked to Caudoviricetes. Ice52 and Ice72 were associated with both Caudoviricetes and Revtraviricetes, while Ice78 was associated with Megaviricetes vOTUs (Table S7).

As we did not obtain any MAGs from microeukaryotes, we investigated their relationships with viruses by identifying similarities between vOTUs and microeukaryotic contigs. For the viral class Megaviricetes, the largest number of similarities was found with the microeukaryotic classes Mucoromycetes (16), Oligohymenophorea (8) and Spirotrichea (3). Regarding the viral class Revtraviricetes, the largest number of similarities was found with Trebouxiophyceae (4), Tremellomycetes (3) and Taphrinomycetes (3) (Table S8). One hundred twenty-three similarities were attributed to unclassified eukaryotes.

### Metagenomic islands

**Fig. 6.**
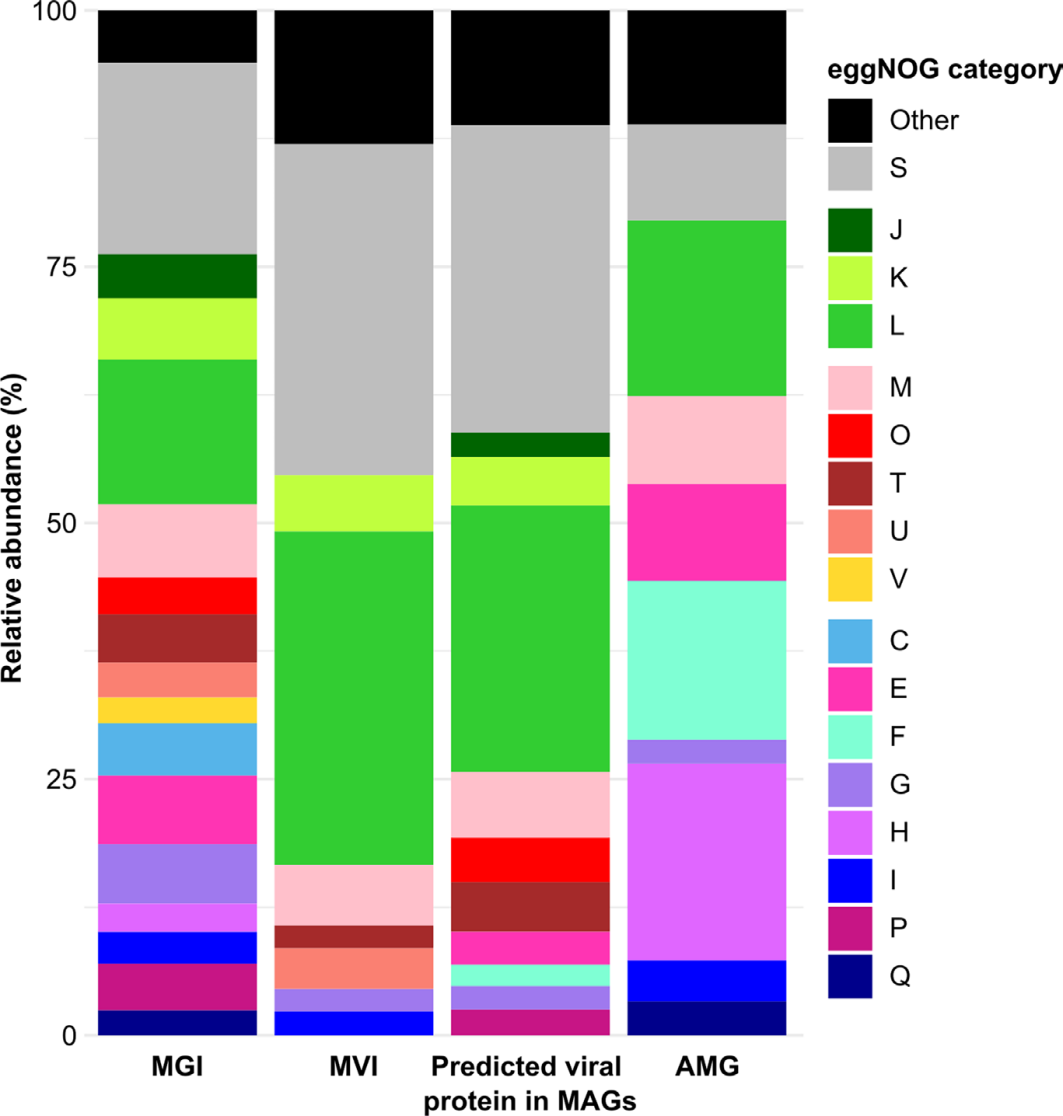
eggNOG categories associated with predicted proteins in MGIs and MVIs, with predicted viral proteins in MAGs and with AMGs. The ‘Others’ category comprises all eggNOG functional categories that were represented by <2% of the predicted proteins. In the figure are represented the eggNOG categories involved in information storage and processing (J: translation, ribosomal structure and biogenesis; K: transcription; L: replication, recombination and repair), cellular processes and signalling (M: cell wall/membrane/envelope biogenesis; O: posttranslational modification, protein turnover and chaperones; T: signal transduction mechanisms; U: intracellular trafficking, secretion and vesicular transport; V: defence mechanisms), metabolism (C: energy production and conversion; E: transport and metabolism; F: transport and metabolism; G: carbohydrate transport and metabolism; H: coenzyme transport and metabolism; I: lipid transport and metabolism; P: inorganic ion transport and metabolism; Q: secondary metabolite biosynthesis, transport and catabolism) and function unknown (S).

We found a total of 12,465 MGIs in the reconstructed MAGs. Of these, 11,235 (90.1%) overlapped with at least 1 predicted CDS. The overlapping CDSs were assigned to 18,304 eggNOGs (Table S9A), and a total of 11,589 CDSs were located within at least 1 MGI. Excluding category S, the most abundant eggNOG category was L (replication, recombination and repair; 14.1% relative abundance), followed by M (cell wall/membrane/envelope biogenesis; 7.2%), E (transport and metabolism; 6.7%) and K (transcription; 6.0%). Sixteen eggNOG categories had relative abundances ≥2% (Fig. 6). The most abundant eggNOGs ascribed to MGIs were involved in viral integration into host DNA (e.g. COG0582 and COG4974), glycosyl group transfer (e.g. COG0438 and COG1215), transposase activity (e.g. COG2801 and COG3385) and colicin transport (e.g. COG1629) (Tables 1a and S10A).

**Table 1.** Number of predicted protein-encoding genes found in MGIs (a) and in MVIs (b) assigned to the 20 most abundant COGs D: cell cycle control, cell division and chromosome partitioning; E: transport and metabolism; G: carbohydrate transport and metabolism; I: lipid transport and metabolism; K: transcription; L: replication, recombination and repair; M: cell wall/membrane/envelope biogenesis; P: inorganic ion transport and metabolism; Q: secondary metabolite biosynthesis, transport and catabolism; S: function unknown; T: signal transduction mechanisms; U: intracellular trafficking, secretion and vesicular transport; V: defence mechanisms; W: extracellular structures.

**(a)**	**eggNOG**	**Category**	**Function**	**Count**
	COG0582	L	DNA integration	121
	COG0438	M	Transferase activity, transferring glycosyl groups	74
	COG1961	L	Site-specific recombinases, DNA invertase Pin homologues	61
	COG4974	L	DNA integration	60
	COG1629	P	Colicin transmembrane transporter	60
	COG0457	S	Peptidyl-tyrosine sulfation	58
	COG2801	L	Transposase and inactivated derivatives	57
	COG0477	EGP	Major facilitator superfamily	53
	COG3385	L	Transposase	51
	COG4771	P	TonB-dependent receptor	45
	COG0286	V	Site-specific DNA-methyltransferase (adenine-specific) activity	43
	COG3335	L	DDE superfamily endonuclease	42
	COG0642	T	Histidine kinase	39
	COG1028	IQ	Dehydrogenase reductase Sdr	38
	COG3209	M	Self proteolysis	36
	COG1215	M	Transferase activity, transferring glycosyl groups	36
	COG0553	L	Helicase	35
	COG2205	T	Histidine kinase, PhoQ sensor	34
	COG1192	D	Chromosome partitioning	34
	COG0451	GM	ADP-glyceromanno-heptose 6-epimerase activity	34
**(b)**	**eggNOG**	**Category**	**Function**	**Count**
	COG0582	L	DNA integration	7
	COG1961	L	Site-specific recombinases, DNA invertase Pin homologues	6
	COG5410	K	Chromosome segregation	5
	COG0270	L	DNA (cytosine-5-)-methyltransferase activity	5
	COG0507	L	Helicase nuclease	4
	COG5362	S	Unknown function	3
	COG3378	KL	Phage plasmid primase P4 family	3
	COG3209	M	Self proteolysis	3
	COG4932	M	Pathogenesis	3
	COG1361	M	Extracellular matrix structural constituent	3
	COG1372	L	Intein-mediated protein splicing	3
	COG2304	IU	Oxidoreductase activity	3
	COG5295	UW	Hep Hag repeat protein	3
	COG1061	L	Helicase	3
	COG2189	L	N(4) N(6)-methyltransferase	3
	COG0457	S	Peptidyl-tyrosine sulfation	3
	COG4570	L	Endodeoxyribonuclease	3
	COG3598	L	Unknown function	2
	1TTJI	L	‘Phage’ integrase family	2
	COG3291	S	Metallopeptidase	2

The MGIs occurred in 92 MAGs, i.e. only Ice46 did not have any MGI. The number of MGIs for the other MAGs ranged from 7 (Ice3 and Ice64) to 1,289 (Ice48). Ice48 was followed by Ice21 (895), Ice60 (647) and Ice68 (639) (Table S11). Of these four MAGs, Ice21, Ice60 and Ice68 presented MGI-predicted proteins mainly ascribed to the eggNOG category L, whereas Ice48 was the only MAG with a prevalence of MGI proteins ascribed to category J (translation, ribosomal structure and biogenesis). Overall, L was the most represented and abundant category in 49 MAGs, followed by E (15 MAGs), M (10) and C (5). Categories G (4), T (4), P (3), U (3), I (2), K (2), H (1) and J (1) were also prevalent in at least one MAG (Fig. S8).

### Metaviromic islands

We detected a total of 2,278 MVIs in 722 (28.1%) of the reconstructed vOTUs. Of these, 443 (17.3%) overlapped with 1 or more CDSs. The corresponding predicted proteins were assigned to 542 eggNOGs (Table S9B). Seven eggNOG classes were present with a relative abundance ≥2% (Fig. 6); therefore, MVIs were represented by fewer functional categories compared with MGIs. Compared with the MGIs, a greater proportion of proteins, in terms of relative abundance, were assigned to category L. Specifically, 32.6% of proteins in MVIs belonged to category L, followed by 5.9% ascribed to M, 5.5% to K, and 3.9% to U (intracellular trafficking, secretion and vesicular transport) (Fig. 6). Many proteins were involved in DNA integration (e.g. COG0582 and COG2189), methyltransferase (e.g. COG0270 and COG2189) and endonuclease (e.g. COG4570) activity (Tables 1b and S10B).

### Viral proteins in MAGs and AMG characterization

Finally, we identified the viral predicted proteins that had correspondences to MAG-predicted proteins. Eighty-four MAGs contained at least 1 viral predicted protein (Table S12), and 2,494 (26.0%) of the hits belonged to eggNOG category L (Fig. 6). Similar trends as for MVIs were observed. We additionally investigated which viral genes from our vOTUs occurred in the MAGs. Most of the genes were ascribed to eggNOG categories involved in transposase activity and DNA integration (Table S13A). The 20 most abundant genes were all assigned to eggNOG category L, except for COG3299, which belongs to the category G (carbohydrate transport and metabolism) (Table S13A).

When we specifically mined for AMGs, we found 162 AMGs from 114 vOTUs. The eggNOG category mainly linked to AMGs was H (coenzyme transport and metabolism; 19.2%), L (17.2%), F (nttransport and metabolism; 15.5%), E (9.6%) and M (8.6%) (Fig. 6). The AMG that was the most abundant was the DNA (cytosine-5-)-methyltransferase activity (COG0270; category L), followed by AMGs involved in ribonucleoside-diphosphate reductase activity (COG0209 with 18 hits and COG0208 with 12 hits; F) (Table S13B). A total of 136 AMGs were found in Caudoviricetes vOTUs, with Duneviridae being the family with the highest number of assigned AMGs (7). Six vOTUs containing AMGs were predicted to infect *Bacteroidota* organisms, five *Pseudomonadota*, four *Actinomycetota*, one *Armatimonadota* and one *Cyanobacteriota* (Table S14).

## Discussion

### Diverse organisms characterize the Rhonegletscher ice microbiome and virome

We observed differences between DNA and RNA trends, with DNA trends reflecting the active, dormant and relic microbial and viral communities, while RNA trends indicated organisms active at the time of sampling. In this context, the observed activity patterns may also reflect the overnight thawing process used to prepare the samples. Therefore, while the community identified through RNA sequences might not have been active on the glacial surface at the time of sampling, we know that they are, at the very least, alive and ready to switch to an active metabolism when the right conditions arise. The observed differences between DNA and RNA patterns may result from the dynamic nature of the weathering crust of a temperate glacier, where the communities were sampled. With its porous ice and high water percolation, this environment undergoes daily and hourly variations due to shifts in sunlight penetration and hydrological changes [15]. This influences microbial activity, as glacial organisms can quickly react to environmental changes, resuming transcriptional activity within 24 h of thawing [19].

The microbial phyla found in our dataset correspond to those often found in supraglacial systems, with organisms belonging to *Pseudomonadota* (e.g. *Acetobacteraceae* and *Polaromonas*), *Bacteroidota* (e.g. *Hymenobacter*), *Actinomycetota* (e.g. *Microbacteriaceae*) and *Cyanobacteriota* (e.g. *Leptolyngbyaceae*) being among the most abundant in both Arctic [19,22] and Alpine [96,97] glaciers. In Rassner *et al*. [16], which investigated the weathering crust by separately analysing its two components (i.e. interstitial porewater and ice matrix), it was observed that the most dominant taxa in these two components were *Polaromonas* and *Hymenobacter*, respectively, which align closely with the patterns observed in our dataset [16]. A high percentage of organisms ascribed to *Vulcanimicrobiota* (a.k.a. WPS-2) can also occur in these systems [19,22], and these organisms were represented at high abundances in our dataset. *Deinococcota* also represented an abundant phylum in our dataset, even if previously highlighted as a rare phylum in glacial environments [22]. *Comamonadaceae* was the most active family from *Pseudomonadota*, representing organisms with diverse metabolisms. The *Comamonadaceae* genera *Polaromonas* and *Variovorax* are represented by chemolithotrophic and hydrogen-oxidizing bacteria and are commonly found in cold and aquatic environments (especially *Polaromonas*); *Variovorax* has been shown to be endophytic [98] and weathering-active on granitic rock in supraglacial environments in the Central Alps [99]. A few MAGs belonging to these families were also ascribed to gas exchange in our analyses (Results S2). In fact, whereas many MAGs were ascribed to heterotrophic organisms, many were also ascribed to organisms able to use reduced compounds and inorganic carbon as energy sources, an important adaptation in oligotrophic environments [100]. The microeukaryotic community also constituted a considerable portion of the glacial biome. The most abundant and active fungi were ascribed to Basidiomycota, Ascomycota and Mucoromycota, probably present on the glacial surface in the form of basidiomycetous yeasts and moulds and commonly found on glacial surfaces [13,19, 96, 101]. The photosynthetic microeukaryotic portion of the community was represented by Chlorophyta, unicellular algae commonly found dominating glacial surfaces [102], and Bacillariophyta, which are diatoms [103]. Additionally, there were other protists, such as Ciliophora, Perkinsozoa and Apicomplexa, which can show parasitic, predatory behaviours towards bacteria, smaller protists and invertebrates [19,27].

In the studied ice samples, bacteriophages were mainly represented by organisms belonging to the classes Caudoviricetes (dsDNA) and Faserviricetes (ssDNA). These organisms and especially Caudoviricetes, the most abundant class in our samples, have already been identified as dominant in glacial environments [25,104]. The high abundance of this bacteriophage class was expected, due to the diverse and complex bacterial communities living on glaciers [105,106]. Interestingly, the high bacterial diversity could also depend on the high diversity of bacteriophages, as the latter are important players in maintaining diversity [33]. The Caudoviricetes family that was most abundant in our dataset was *Peduoviridae*, whereas the most active one was *Autographiviridae*; these taxa have already been found in proglacial waters [107].

Eukaryotic viruses were also well represented in our dataset and are known to modulate the metabolism of microeukaryotes and have mutualistic interactions with them [108]. Revtraviricetes was the most abundant viral class in the RNA dataset, with high transcription shown for the family *Caulimoviridae*, represented by dsDNA viruses mainly infecting plants [109]. Another family represented in the dataset, though with lower abundance, was the RNA virus *Retroviridae*, mainly infecting vertebrates [110]. Megaviricetes are a class of DNA giant viruses with large genomes, containing a large number of genes involved in DNA repair, transcription, replication and translation compared with other viral classes [111]. They are known to infect eukaryotes: for example, *Mimiviridae* infect aquatic eukaryotes, especially amoeba [112]; *Iridoviridae* infect vertebrates and arthropods [113]; and *Phycodnaviridae* infect marine and freshwater algae [114]. Organisms belonging to Megaviricetes, associated with algae and protists, have also been detected previously in ice environments [25,28].

### Virus–host associations and their specificity

Viral dynamics influence the microbial populations (and vice versa) in various environments [115], including supraglacial systems [34,35]. The dynamics between viral and microbial communities strongly depend on the environmental and population settings [30,116], but in general, viruses tend to preferentially infect metabolically active organisms [117]. Most viruses in our dataset were in the lytic phase, which is expected under favourable conditions in oligotrophic environments [31,32, 36]. This matches our observations that the lytic lifestyle is prevalent in the weathering crust during the ablation season, when nutrients and water are more available and temperatures are higher than in the accumulation season. On the contrary, viruses in 15,000-year-old ice cores from the Tibetan Plateau were shown to be in a lysogenic phase [42], indicating that viruses might enter the lysogenic phase when only basal metabolism is likely to be maintained in the host cells [118]. It is important to acknowledge the limitations of the current approach in distinguishing between lytic and lysogenic viruses, as it relies on detecting lysogenic marker proteins which depend heavily on genome completeness.

When we analysed virus–prokaryote infectivity interactions, we observed that most viruses belonged to the main bacteriophage class Caudoviricetes [119]. Viruses in this class infected organisms belonging to a variety of bacterial classes, which was expected given the high taxonomic ranks considered. Mavrich and Hatfull [120] showed that some viruses are specific to a genus, while others are not [120]. This was evident in our dataset when we looked at virus–MAG correspondences: some vOTUs showed specificity at the family level, some at the genus level and some even at the MAG level. There were only a few signatures of bacterial infection by one vOTU across classes or phyla. Additionally, three MAGs (Ice9, Ice32 and Ice48) had smaller genomes (<2 Mb) compared with the others and had no matches with vOTUs. Ice32 and Ice48, belonging to the CPR group, are probably symbiotic bacteria [121], and *Candidatu*s *Nanopelagicaceae* (Ice19) is a common organism in water environments and is auxotrophic [122]; these organisms, which lack several genes essential for independent growth, might also be unsuitable for viral infection. Furthermore, organisms from the CPR group may escape infection by deleting common membrane phage receptors [121]. Taxa with more than one MAG, where no viral infection signatures were found in any of them, belonged to *Chloroflexota* and *Rhodanobacteraceae* (within the phylum *Pseudomonadota*). This result may be influenced by the use of metagenomically reconstructed bacterial and viral genomes, which could contain errors and be incomplete. However, direct studies specifically focused on viruses infecting these organisms remain limited, and no information is currently available on how these organisms might evade viral infection.

Other viral classes shown to infect prokaryotic organisms included Megaviricetes, which are eukaryotic giant viruses. Megaviricetes are known to carry genes derived from prokaryotic organisms, even though they do not directly infect prokaryotes [123,124]. Further highlighting viral interactions across all levels, with genes and genomes interlaced even among different life domains, we observed that the main eukaryotes interacting (i.e. having matching gene signatures) with the two primary classes of eukaryotic viruses (Megaviricetes and Revtraviricetes) were the green algae class Trebouxiophyceae, the fungal classes Mucoromycetes, Tremellomycetes and Eurotiomycetes and the ciliate class Oligohymenophorea. All of these eukaryotes have been previously found in snow, glacial or early-stage forefield environments [11,14, 96, 101, 102, 125].

### MVIs and MGIs

Considering both MVIs and MGIs can provide key information about population variability. In our dataset, MGIs were predominantly ascribed to 16 eggNOG categories, whereas MVIs were only ascribed to 7, demonstrating a wider range of functionalities within MGIs compared with MVIs. This result reflects the fact that MVIs in viruses are attributed to the main function of evading host cell responses [38,126], whereas MGIs in micro-organisms have been linked to a wider range of functions [40,127].

The main functions that were ascribed to MVIs were those connected to categories of replication, recombination and repair (L), transcription (K) and cell wall/membrane/envelope biogenesis (M). These functions are all potentially involved in viral replication in the host cells and are therefore easily recognizable by the hosts. In particular, many of the predicted genes encoded viral endonucleases, integrases, recombinases and transposases. Endonucleases allow viruses to insert and replicate in the host genome [128]. Integrases and recombinases enable viruses to catalyse site-specific recombination between the bacterial genome and foreign DNA [129,130]. Transposases, which made up most of the detected genes, are usually found in host genomes and ligate at the borders of transposons to move genes and may also help hosts defend against viral infections. Transposons have been found to be upregulated in host cells after viral infection, indicating a role for these mobile elements in host cell responses to viruses [131]. Furthermore, transposable elements have been shown to play various roles in bacteria, including defensive functions [132]. Genes encoding transposases [2] are among the most abundant genes in all genomes across all domains [133]. Because these genes are important for genome regulation, they are probably subject to constant horizontal gene transfer across domains. The similarity between genes within MGIs and MVIs in our study suggests considerable gene exchange between viruses and host cells. This was further supported by our investigation of viral proteins present in host cells. These, once again, could be ascribed to eggNOG category L, indicating that these genes may be remnants of past or active infections. This is important, as virulence factors have been found in bacteria in glacial systems [22] and can be transmitted between viruses and bacteria [134].

Most of the bacterial MGIs were ascribed to the functional categories L, K and M, and also to metabolic pathway categories such a transport and metabolism (E), energy production and conversion (C) and carbohydrate transport and metabolism (G). These categories are also ascribed to stress adaptations [135,136] and microbial competition. In fact, MGIs contained genes encoding colicin transport proteins (involved in secreting and importing antimicrobial compounds) and Rhs proteins (mediating intercellular competition) [137,138].

In our dataset, we also observed signatures of possible viral modulation of host cell metabolism, as previously observed in glacier-related environments [25], and AMGs have previously been ascribed to glacial viruses [38]. Most AMGs were ascribed to the eggNOG category H (coenzyme transport and metabolism), indicating that viruses might be able to modify host metabolic pathways related to coenzyme biosynthesis, uptake or utilization (e.g. NAD, FAD and CoA) to support infection and replication. The most abundant COGs were ascribed to methylases, enzymes involved in the catalysis of RNA methylation, which might therefore help viruses enhance host metabolic activity, evade immune responses or regulate viral replication [139].

However, it is important to acknowledge that virus–host interactions, as well as MVIs, MGIs and AMGs, were inferred solely through *in silico* analyses and have not been experimentally validated.

## Conclusions

Our dataset, comprising only four samples taken at a single time point, is not fully representative of the entire Rhonegletscher or of the microbiome’s variability over time and therefore does not allow statistically robust conclusions. However, this study provides the first insights into the glacier’s microbiome, revealing a diverse community of prokaryotic, microeukaryotic and viral organisms, whose dominant taxa were also reported on other glaciers. The community also showed activity patterns that do not strictly correspond to the DNA signatures of the microbial pool present in the system. This suggests that the glacial microbiome consists of a diverse pool of organisms, with activity that could be influenced by varying glacial conditions. Notably, the dynamic of these glacial microbiomes is also reflected in the genomic signatures observed in the host–virus relationships, where gene flux between these components indicated dynamic genetic exchange. The variability of genes involved in host–virus recognition and stress adaptations, evidenced by the presence of MVIs and MGIs, further highlights the complex, interlaced relationships within these communities, including the presence of giant viruses with prokaryotic signatures. These findings underscore the intricate and evolving nature of glacial communities, where the dynamics between prokaryotes, eukaryotes and viruses, whose activity is influenced by glacial conditions, are likely to change due to global warming. Such shifts in the glacial biome will also alter downstream ecosystems worldwide as active viruses and bacteria are released, maintaining infectivity and adaptability due to the persistence of MVIs and MGIs. This could significantly impact downstream communities, altering nutrient and carbon cycling processes through changes in viral shunt mechanisms and biome activities.

## Supplementary material

10.1099/mgen.0.001524 Uncited Supplementary Material 1.

10.1099/mgen.0.001524 Uncited Supplementary Material 2.

## Abbreviations

AMG: auxiliary metabolic gene
CDS: coding sequence
CPR: candidate phyla radiation
MAG: metagenome-assembled genome
MGI: metagenomic island
MVI: metaviromic island
TPM: tags per million
vOTU: viral operational taxonomic unit
