# Erratum: Host–virome associations in the weathering crust of a rapidly retreating temperate Alpine glacier

**DOI:** 10.1099/mgen.0.001619

**Published:** 2026-01-20

**Authors:** Gilda Varliero, Andreas Bauder, Beat Stierli, Weihong Qi, Beat Frey

**Affiliations:** 1 Rhizosphere Processes Group, Swiss Federal Institute for Forest, Snow and Landscape Research (WSL), Birmensdorf, Switzerland; 2 Laboratory of Hydraulics, Hydrology and Glaciology (VAW), ETH Zurich, Zurich, Switzerland; 3 Swiss Federal Institute for Forest, Snow and Landscape Research (WSL), Sion, Switzerland; 4 Functional Genomics Center Zurich, ETH Zurich and University of Zurich, Zurich, Switzerland; 5 Swiss Institute of Bioinformatics SIB, Geneva, Switzerland

In the published version of this article, the terms ‘nucleotide’ and ‘amino acid’ were omitted from several descriptions of eggNOG functional categories.

The instances in which the terms were omitted are as follows:

- In the section ‘Viral proteins in MAGs and AMG characterization’, eggNOG category F has been updated to read ‘nucleotide transport and metabolism’.
- In the section ‘Metagenomic Islands’, eggNOG category E has been updated to read ‘amino acid transport and metabolism’.
- In the section ‘MVIs and MGIs’, references to eggNOG category E have been updated to read ‘amino acid transport and metabolism’.
- In the legend of Figure 6, eggNOG categories E and F have been updated to read ‘amino acid transport and metabolism’ and ‘nucleotide transport and metabolism’, respectively.
- In the legend of Table 1, eggNOG category E has been updated to read ‘amino acid transport and metabolism’.

The published article has been updated to reflect these corrections.

The Microbiology Society apologise for any confusion or inconvenience caused.

