# Supplementary material for: Host–virome associations in the weathering crust of a rapidly retreating temperate Alpine glacier
Source: Microb Genom. 2025 Oct 3;11(10):001524. doi: 10.1099/mgen.0.001524 (PMC12494485; doi:10.1099/mgen.0.001524)
Supplement: Uncited Supplementary Material 1. [file mgen-11-01524-s001.pdf]

Supplementary Materials

Host–virome associations in the weathering crust of a rapidly retreating temperate Alpine glacier

Gilda Varliero, Andreas Bauder, Beat Stierli, Weihong Qi, Beat Frey

Figure S1. Location of the Rhonegletscher in Switzerland (A), photograph of the glacier (B), map of the glacier highlighting the area where the three sampling sites were located in its ablation zone (C), and two bags containing the collected ice (D).

Figure S2. Bioinformatics pipeline used in the study.

Figure S3. DNA (A) and RNA (B) base coverage of metagenome-assembled genomes (MAGs). The base coverage is defined here as the average number of DNA or RNA nucleotide bases aligned to a specific locus in an MAG. MAG names with lower numbers correspond to MAGs with a higher genome completeness (e.g., "Ice1" is the MAG with the highest completeness).

Figure S4. Viral operational taxonomic unit (vOTU) sequence length distribution.

Figure S5. Virus relative abundance for the DNA datasets at the family level (A), along with the percentages of virulent and temperate viruses associated with each family (B). Classification of families by class (C). Relative abundances are represented as TPM (tags per million) transformed as $\log_{10}$(TPM + 1). The median and quartiles for each family are displayed in the box plots (n = 4).

Figure S6. Virus relative abundance for the RNA datasets at the family level (A). Classification of families by class (B). Relative abundances are represented as TPM (tags per million) transformed as $\log_{10}$(TPM + 1). The median and quartiles for each family are displayed in the box plots (n = 4).

Figure S7. Number of viral operational taxonomic units (vOTUs) classified at the family level associated with bacterial classes (A) and families (B). Viral families associated with only one bacterial taxon are not included in the plot.

Figure S8. Scaled abundance of predicted proteins ascribed to metagenome-assembled genomes (MAGs) and classified with the eggNOG classification. R (general function prediction only) and S (function unknown) categories are not reported. White dots indicate the dominant category (categories) in each metagenome-assembled genome (MAG). C: energy production and conversion; D: cell cycle control, cell division and chromosome partitioning; E: amino acid transport and metabolism; F: nucleotide transport and metabolism; G: carbohydrate transport and metabolism; H: coenzyme transport and metabolism; I: lipid transport and metabolism; J: translation, ribosomal structure and biogenesis; K: transcription; L: replication, recombination and repair; M: cell wall/membrane/envelope biogenesis; N: cell motility; O: post-translational modification, protein turnover and chaperones; P: inorganic ion transport and metabolism; Q: secondary metabolite biosynthesis, transport and catabolism; T: signal transduction mechanisms; U: intracellular trafficking, secretion and vesicular transport; V: defence mechanisms; W: extracellular structures; Y: nuclear structure; Z: cytoskeleton.

Supplementary tables S1 to S14. These are reported in an external excel file.

Supplementary Results 1. Ice chemistry.

Supplementary Results 2. Predicted metabolic energy production pathways and adaptations to the glacial environment of the metagenome-assembled genomes (MAGs).

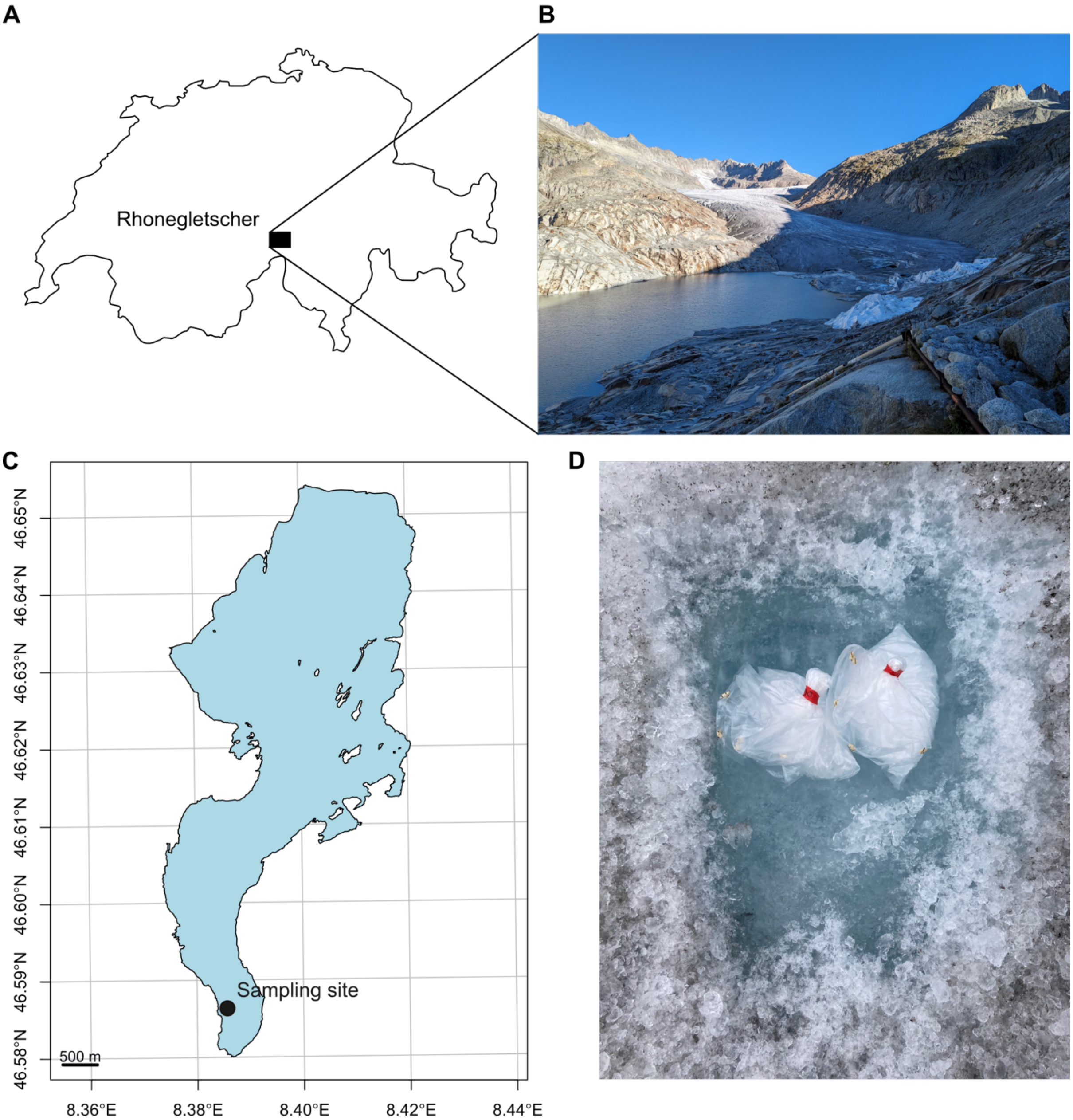

Figure S1. Location of the Rhonegletscher in Switzerland (A), photograph of the glacier (B), map of the glacier highlighting the area where the three sampling sites were located in its ablation zone (C), and two bags containing the collected ice (D).

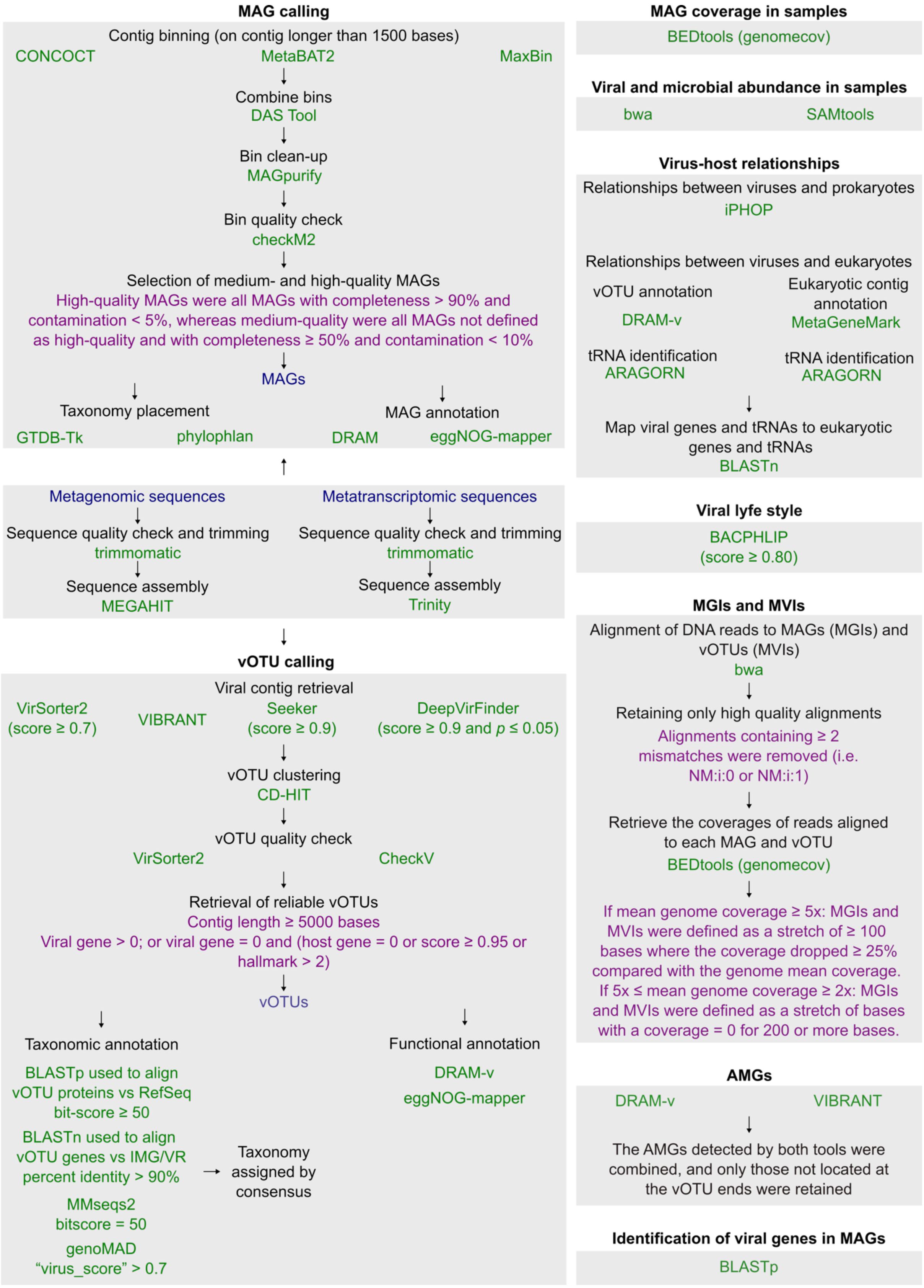

Figure S2. Bioinformatic pipeline used in the study.

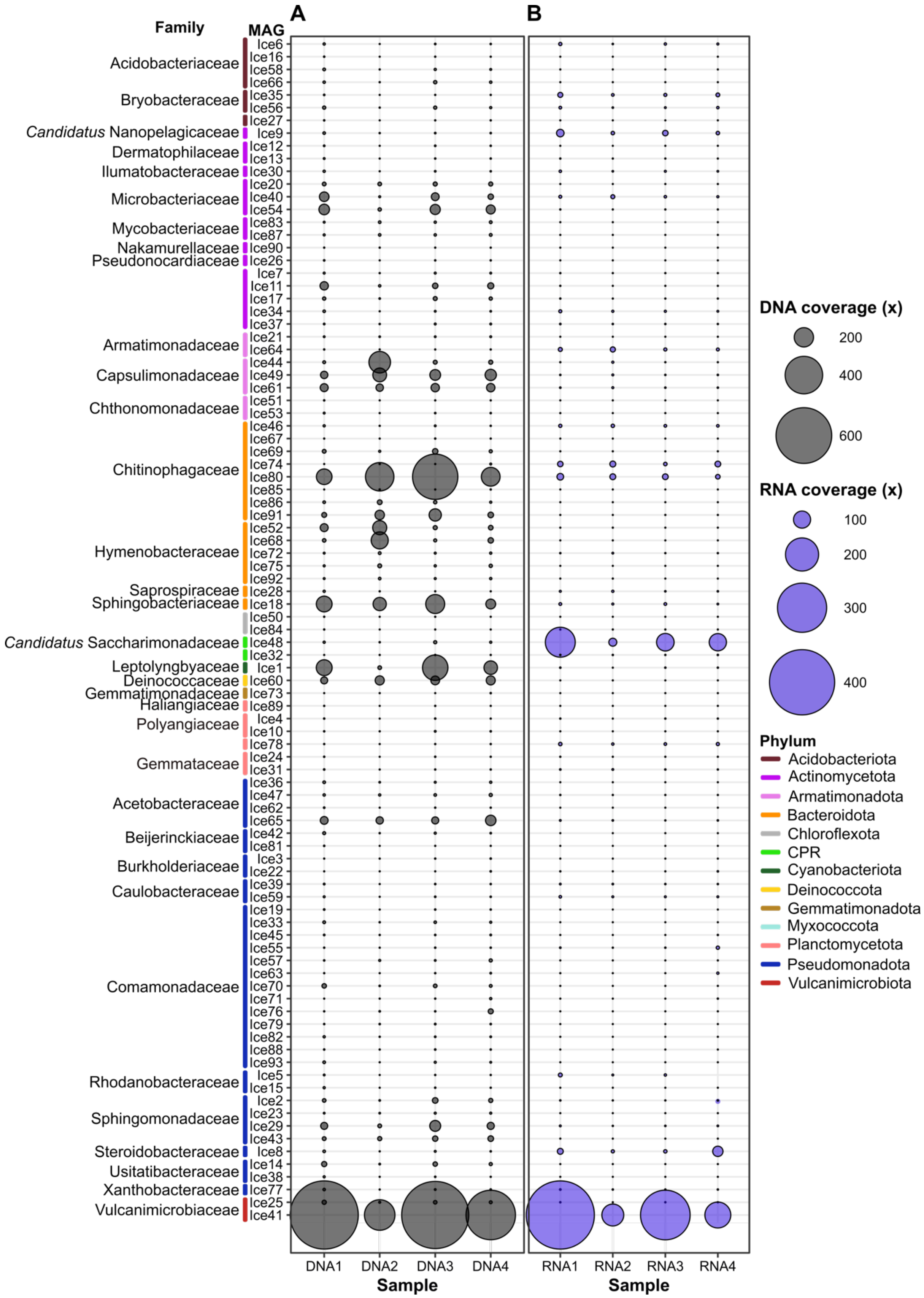

Figure S3. DNA (A) and RNA (B) base coverage of metagenome-assembled genomes (MAGs). The base coverage is defined here as the average number of DNA or RNA nucleotide bases aligned to a specific locus in an MAG. MAG names with lower numbers correspond to MAGs with a higher genome completeness (e.g., "Ice1" is the MAG with the highest completeness).

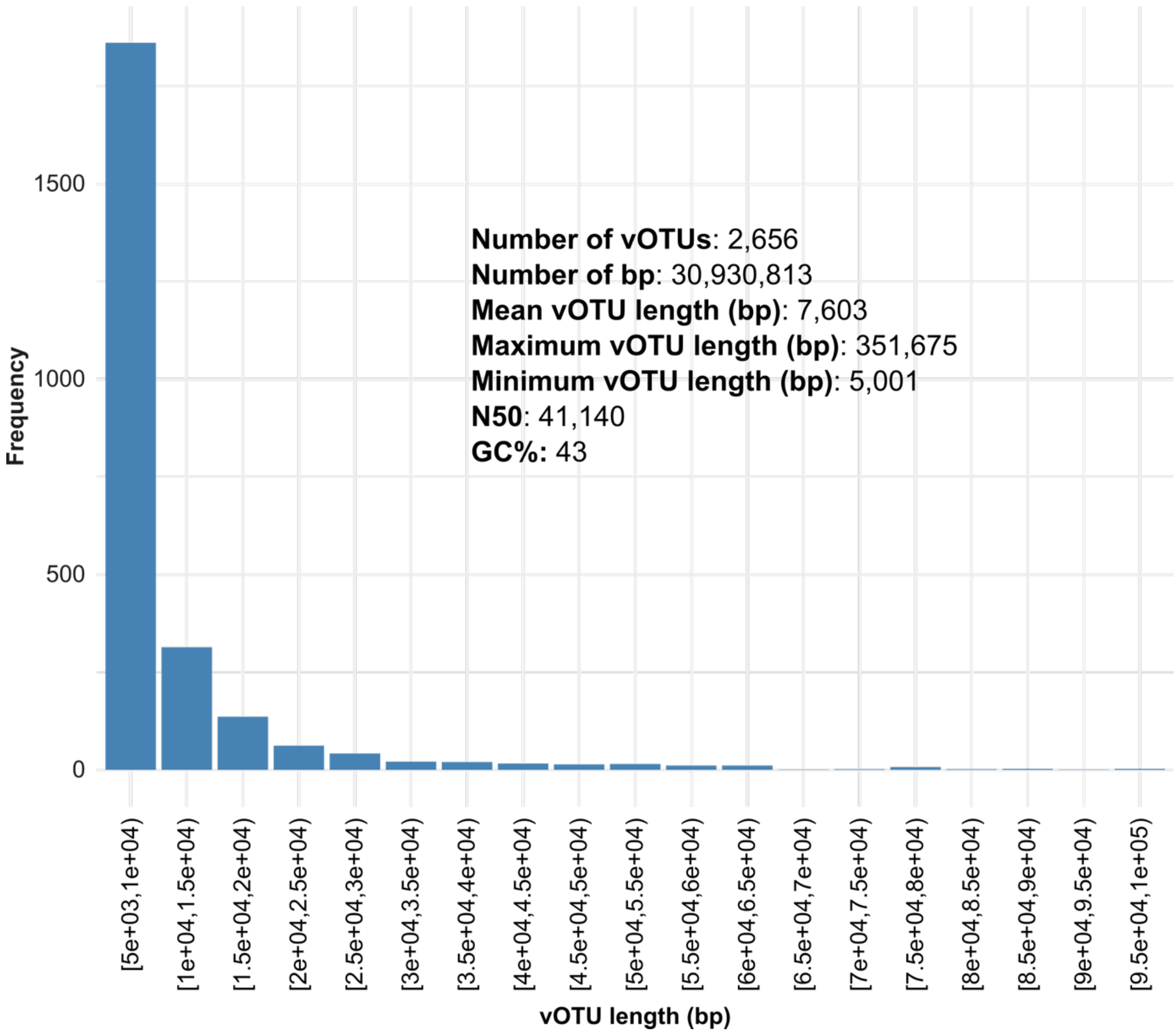

Figure S4. Viral operational taxonomic unit (vOTU) sequence length distribution.

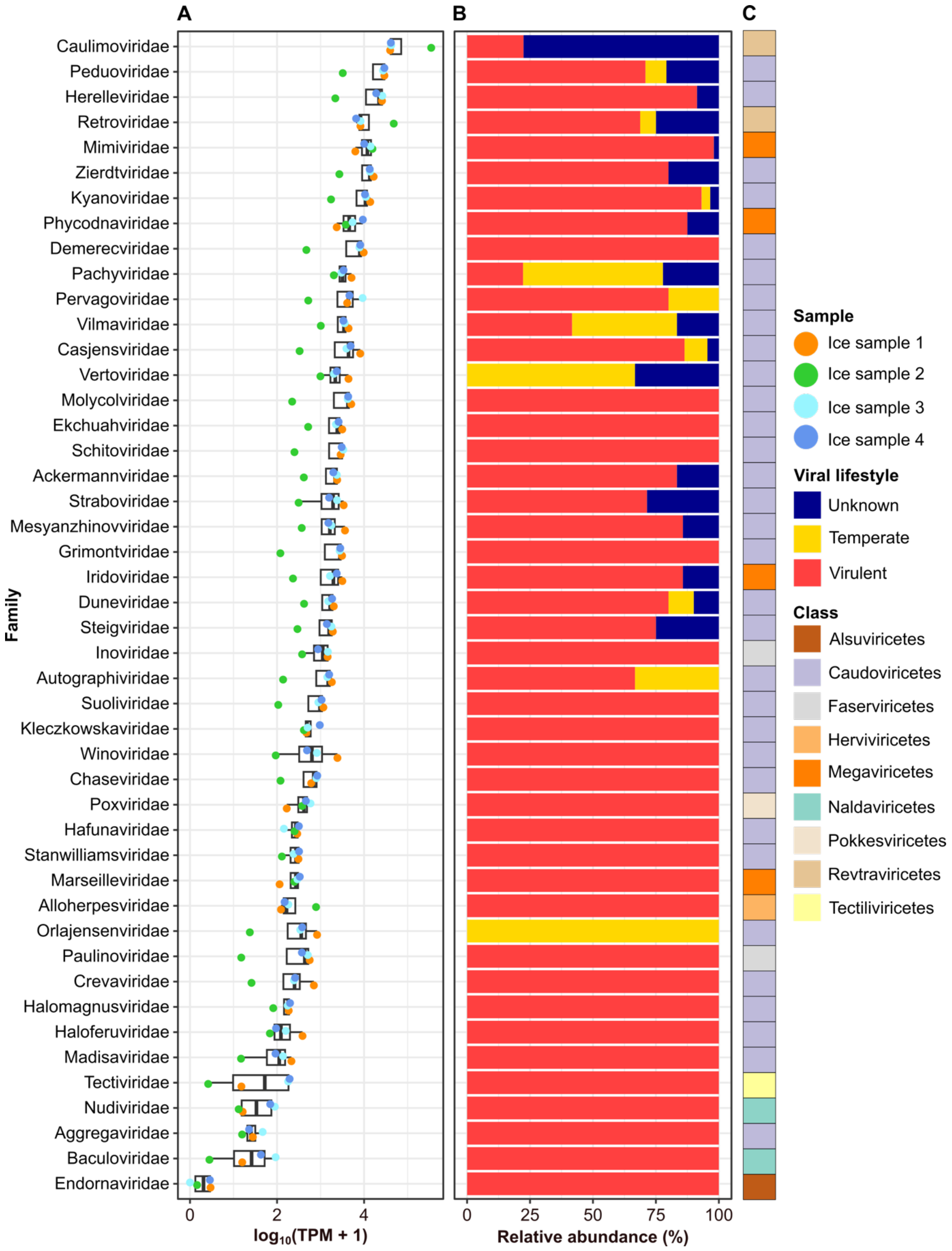

Figure S5. Virus relative abundance for the DNA datasets at the family level (A), along with the percentages of virulent and temperate viruses associated with each family (B). Classification of families by class (C). Relative abundances are represented as TPM (tags per million) transformed as $\log_{10}(\text{TPM} + 1)$. The median and quartiles for each family are displayed in the box plots ($n = 4$).

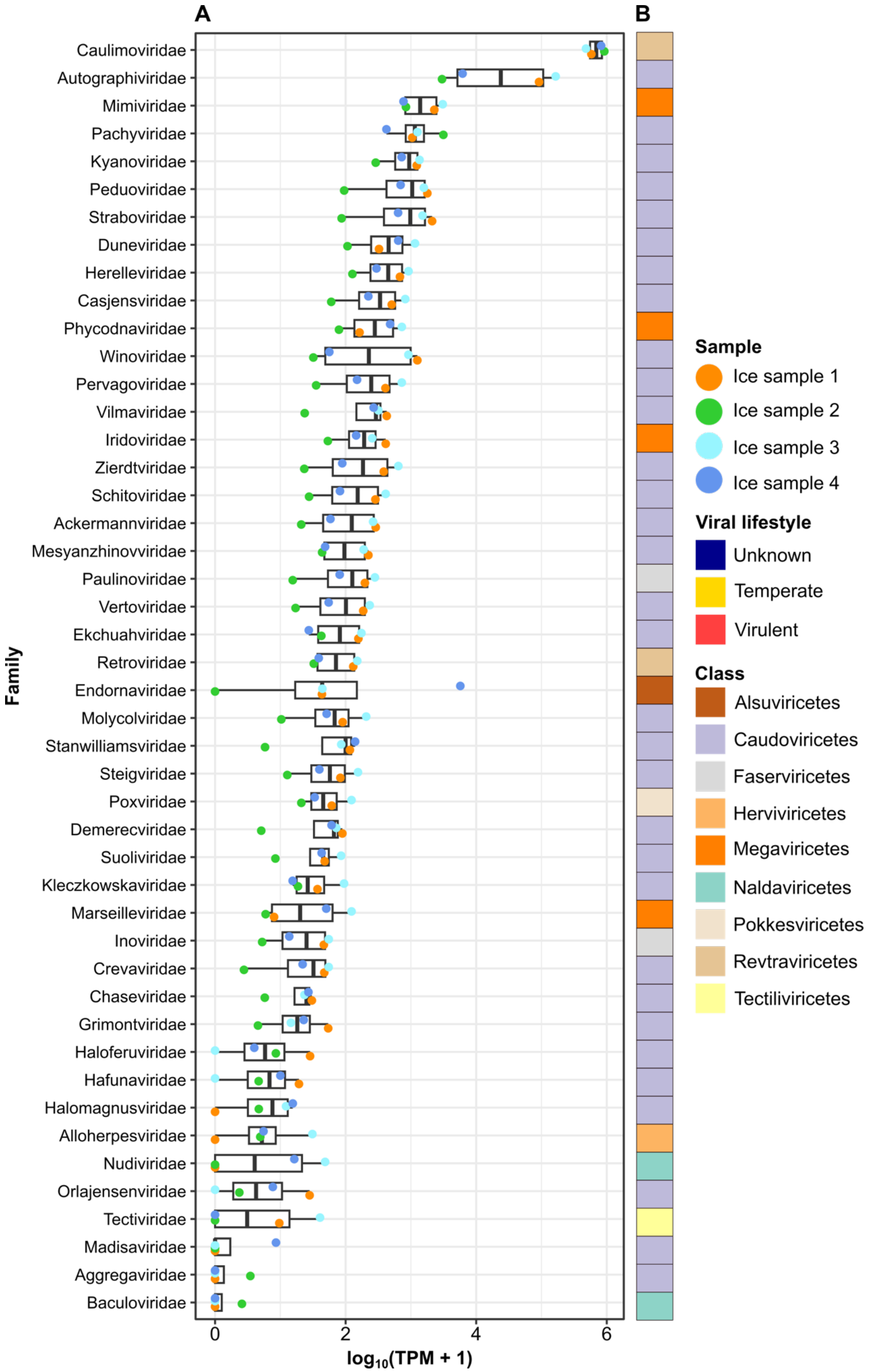

Figure S6. Virus relative abundance for the RNA datasets at the family level (A). Classification of families by class (B). Relative abundances are represented as TPM (tags per million) transformed as $\log_{10}$(TPM + 1). The median and quartiles for each family are displayed in the box plots (*n* = 4).

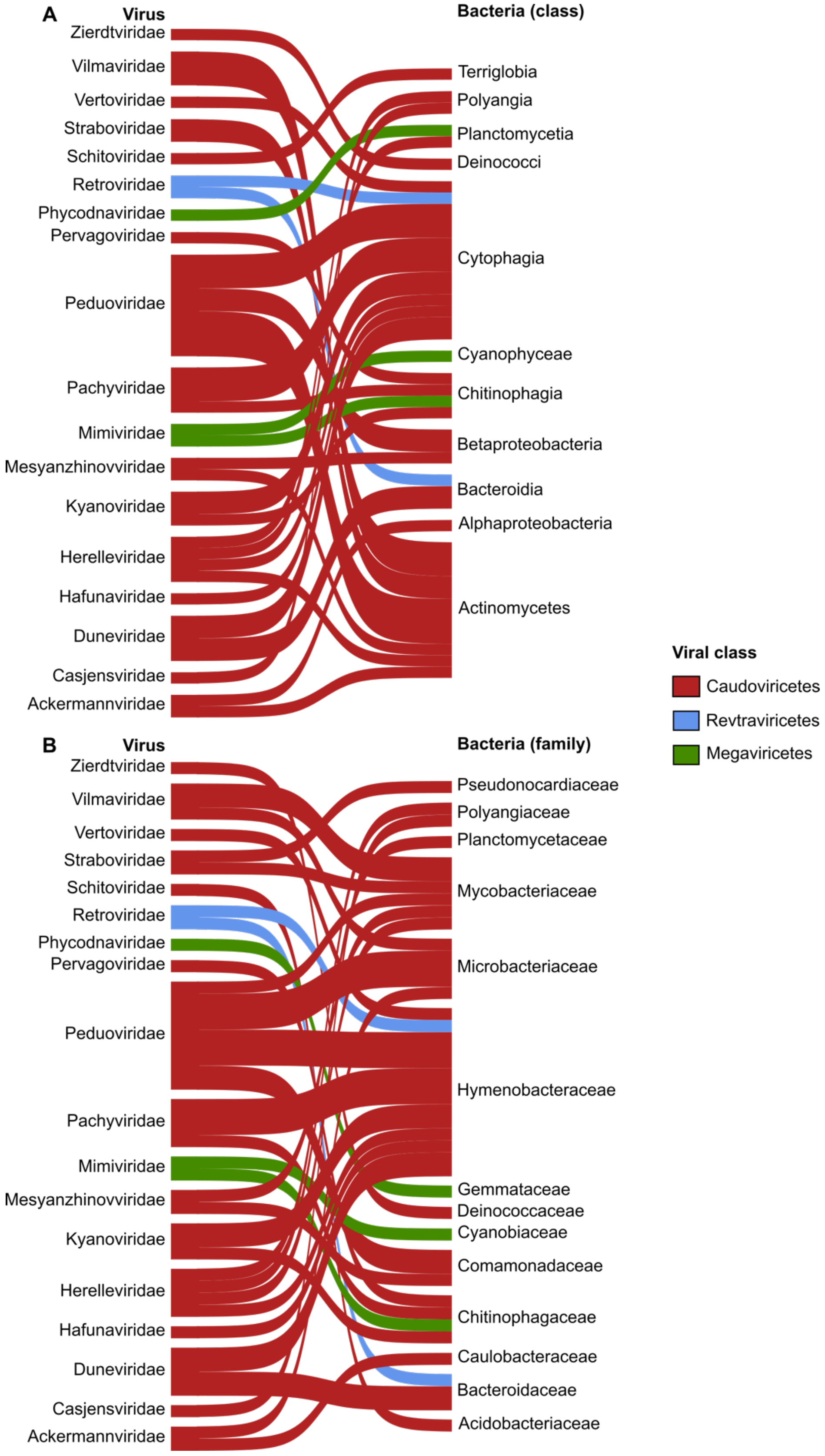

Figure S7. Number of viral operational taxonomic units (vOTUs) classified at the family level associated with bacterial classes (A) and families (B). Viral families associated with only one bacterial taxon are not included in the plot.

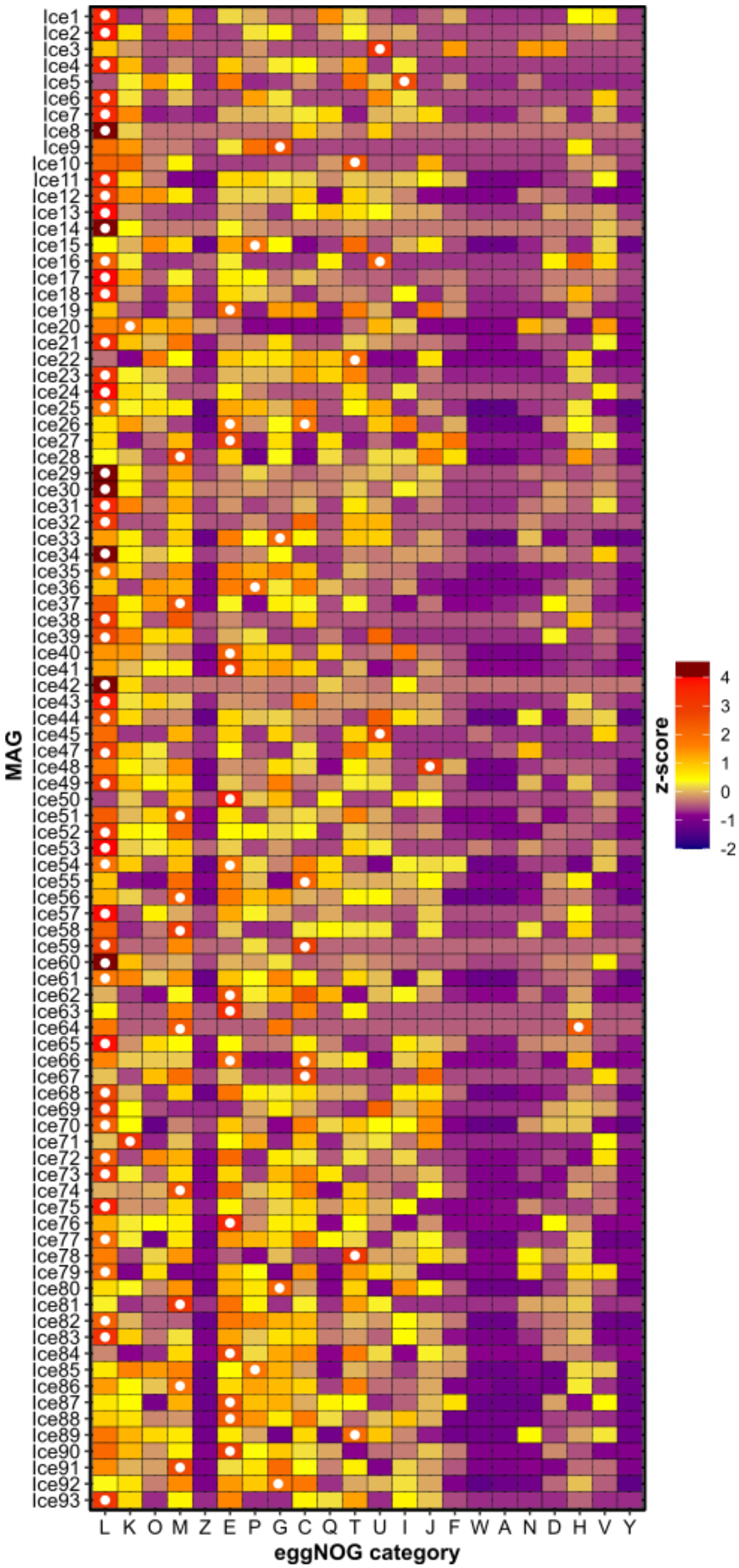
MAG
eggNOG category
L K O M Z E P G C Q T U I J F W A N D H V Y
z-score
4
3
2
1
0
-1
-2

Figure S8. Scaled abundance of eggNOG-classified predicted proteins that fell into the metagenomic islands (MGIs) found in the metagenome-assembled genomes (MAGs). R (general function prediction only) and S (function unknown) categories are not reported. White dots indicate the dominant category (categories) in each metagenome-assembled genome (MAG). C: energy production and conversion; D: cell cycle control, cell division and chromosome partitioning; E: amino acid transport and metabolism; F: nucleotide transport and metabolism; G: carbohydrate transport and metabolism; H: coenzyme transport and metabolism; I: lipid transport and metabolism; J: translation, ribosomal structure and biogenesis; K: transcription; L: replication, recombination and repair; M: cell wall/membrane/envelope biogenesis; N: cell motility; O: post-translational modification, protein turnover and chaperones; P: inorganic ion transport and metabolism; Q: secondary metabolite biosynthesis, transport and catabolism; T: signal transduction mechanisms; U: intracellular trafficking, secretion and vesicular transport; V: defence mechanisms; W: extracellular structures; Y: nuclear structure; Z: cytoskeleton.

Supplementary Results 1

The pH of the melted glacial ice was 6.61 ± 0.11, $\delta^{18}O$ was -15.17 ± 1.53‰, δD was -105.65 ± 11.52‰, chloride concentration was 0.019 ± 0.003 µg L^{-1} and nitrate concentration was 0.037 ± 0.001 µg L^{-1}. Nitrite (limit of detection (LOD) = 0.010 µg L^{-1}), phosphate (LOD = 0.150 µg L^{-1}) and sulphate (LOD = 0.050 µg L^{-1}) concentrations were below the detection limit (Supplementary Results Table 1).

Supplementary Results Table 1. Ice chemistry. Chloride limit of detection (LOD) = 0.010 µg L^{-1}; Nitrite LOD = 0.010 µg L^{-1}; Nitrate LOD = 0.035 µg L^{-1}; Phosphate LOD = 0.150 µg L^{-1}; Sulphate LOD = 0.050 µg L^{-1}.

Sample	pH	$\delta^{18}O$ ‰	δD ‰	Chloride µg L^{-1}	Nitrite µg L^{-1}	Nitrate µg L^{-1}	Phosphate µg L^{-1}	Sulphate µg L^{-1}
Ice sample 1	6.56	-13.4	-93.7	0.022	<0.01	0.038	<0.15	<0.05
Ice sample 2	6.71	-15.4	-108.1	0.018	<0.01	0.035	<0.15	<0.05
Ice sample 3	6.69	-17.1	-120.5	0.021	<0.01	0.037	<0.15	<0.05
Ice sample 4	6.47	-14.8	-100.3	0.016	<0.01	0.038	<0.15	<0.05

Supplementary Results 2

We also explored the metabolism of metagenome-assembled genomes (MAGs) to understand their energy production pathways and adaptations to the glacial environment. This was accomplished by identifying predicted proteins involved in energy metabolisms and adaptations to the glacial environment (Photosystem I: *psaA* and *psaB*. Photosystem II: *psbA*, *psbB*, *psbD*, *psbH*, *PufL* and *PufM*. Hydrogenase: *hynA*, *hynB*, *hynI*, *HydA*, *HydD* and *HydF*. Nitrogenase: *nifH*. Carbon monoxide dehydrogenase: *CoxL*. CO_2 fixation [Calvin cycle]: *rbcS* and *rbcL*. CO_2 fixation [others]: *aclA*, *mcmA* and *AcsB*. Ferredoxins: *petF*, *petH*, *fdxA*, *fdxH*, *fdxN*, *fldA*, *hyaB*, *narH* and *petJ*. Pigments: *chlL*, *bchD*, *bchF*, *bchN*, *bchX*, *bchY*, *bchZ*, *crtB*, *crtL* and *crtE*. Photolyase: *phrA* and *phrB*. EPS: *epsA*, *epsB*, *epsE* and *epsR*. Movement and chemotaxis: *fliC*, *matA*, *cheA*, *tsr*, *pilA*, *pilB*, *pilH*, *flp*, *PulO* and *mcp*. Branched-chain alpha-keto acid dehydrogenase complex [BCKDC]: *bkdA*, *bkdB*, *bkdC* and *bkdR*)[1,2]. Protein sequences were downloaded from UniProt[3] [20/05/2024] by searching for the gene name in the database search engine and filtering the results only for the bacterial domain. These proteins were then aligned to the predicted MAG proteins using diamond v2.1.8.162[4] with the parameters "--id 50 --query-cover 80".

Thirty-three MAGs matched proteins from Photosystem I, whereas nine matched proteins from Photosystem II. Several bacteria also showed proteins potentially involved in the

metabolism of trace gases, such as H_2, N_2 and CO. Twenty-six MAGs matched proteins involve in the Calvin cycle. MAGs had a variety of ferredoxins, with most of the hits were obtained for the gene *fdxA*, encoding the 2[4Fe–4S] ferredoxin. We then tested for adaptations, such as pigment production, photorepair processes, exopolysaccharide production, chemotaxis and synthesis of branched-chain keto acids. All MAGs showed predicted genes in at least two of these categories. The most widespread adaptation in the MAGs was photorepair, where only eight MAGs did not match the investigated proteins (i.e. CPD photolyase and (6-4) photolyase). Ice41, the most abundant and active MAG in our dataset, showed predicted proteins related to Photosystem II, ferredoxin *fdxA*, bacteriochlorophyll synthesis, photolyases, EPS production, type IV major pilin, and the branched-chain alpha-keto acid dehydrogenase complex (BCKDC) (Supplementary Results Fig. 1; Supplementary Results Table 2).

Our analysis indicated genes for these metabolism pathways in organisms not previously ascribed to them, demonstrating how widely these metabolic traits are spread across the environment. For example, a few MAGs were ascribed to photosynthetic families such as Leptolyngbyaceae[5], or to Gemmatimonadaceae and Comamonadaceae where photosynthetic organisms have been identified[6,7]. Detected organisms able to play a role in atmospheric trace gas (e.g. H_2 and CO) or N_2 uptake belonged to Chloroflexia, Vulcanimicrobiaceae, Armatimonadia, Beijerinckiaceae, Xanthobacteraceae, Burkholderiaceae and Comamonadaceae, where chemosynthetic carbon fixation was also attributed to these taxa. Finally, organisms in the taxa Myxococcota, Beijerinckiaceae, Xanthobacteraceae, Rhodanobacteraceae, Burkholderiaceae, Comamonadaceae and Leptolyngbyaceae are known to perform mixotrophy, where organic or inorganic compounds can be used as a carbon source depending on the environmental conditions[8].

The microbiome of the ice sampled from the Rhonegletscher seems to be well adapted to the glacier conditions, as the MAGs, representative of a large portion of the glacial microbiome, encoded proteins (also actively transcribed) that enable them to handle the various challenges that are posed by this environment[1,9]. Among these were photolyases, i.e. enzymes used to repair DNA damage caused by UV light, which is intense on the glacial surface[10,11]. Pigments, essential for photosynthetic pathways, can also provide protection from UV light[12,13] and were represented in the MAGs. Genes encoding proteins involved in extracellular polymeric substance (EPS) production, which is used for cryoprotection, were also present, along with those for the branched-chain α-keto acid dehydrogenase complex (BCKDC), which catalyses the oxidative decarboxylation of branched-chain α-keto acids, producing precursors for branched-chain fatty acids that help maintain membrane fluidity in cold environments[14].

Finally, we found that a substantial portion of the microbiome could synthesise proteins involved in motility and chemotaxis, functions that are especially important in oligotrophic environments such as glaciers[15].

Supplementary Results Table 2. Proteins retrieved from UniProt (https://www.uniprot.org) to investigate the metabolism of metagenome-assembled genomes (MAGs).

Process	Gene	Encoded protein	Further information
Photosynthesis	*psaA*	Photosystem I P700 chlorophyll a apoprotein A1	Photosystem I
Photosynthesis	*psaB*	Photosystem I P700 chlorophyll a apoprotein A2	Photosystem I
Photosynthesis	*psbA*	Photosystem II protein D1	Photosystem II
Photosynthesis	*psbB*	Photosystem II CP47 reaction center protein	Photosystem II
Photosynthesis	*PufL*	Reaction center protein L chain	Photosystem II
Photosynthesis	*PufM*	Reaction center protein M chain	Photosystem II
Photosynthesis	*psbD*	Photosystem II D2 protein	Photosystem II
Photosynthesis	*psbH*	Photosystem II reaction center protein H	Photosystem II
Hydrogenases	*NiFe*	nitrogenase molybdenum-cofactor synthesis protein	NiFe hydrogenase
Hydrogenases	*hynA*	cytochrome-c3 hydrogenase	NiFe hydrogenase
Hydrogenases	*hynB*	Periplasmic [NiFe] hydrogenase small subunit 1	NiFe hydrogenase
Hydrogenases	*hynl*	Uptake hydrogenase large subunit	NiFe hydrogenase
Hydrogenases	*HydA*	Periplasmic [Fe] hydrogenase large subunit	Fe hydrogenase
Hydrogenases	*hydF*	[FeFe] hydrogenase H-cluster maturation GTPase	Fe hydrogenase
Hydrogenases	*hydD*	Hydrogenase 2 maturation protease	Fe hydrogenase
Nitrogenases	*nifH*	Iron protein subunit of hydrogenase	Nitrogenase
Atmospheric CO uptake	*CoxL*	Carbon monoxide dehydrogenase	
CO2 fixation (Calvin cycle)	*rbcL*	ribulose-1,5-bisphosphate carboxylase/oxygenase large subunit	Calvin-Benson-Bassham Cycle
CO2 fixation (Calvin cycle)	*rbcS*	Ribulose bisphosphate carboxylase small subunit	Calvin-Benson-Bassham Cycle
CO2 fixation (others)	*aclB*	ATP-citrate lyase beta-subunit	Reductive TCA cycle
CO2 fixation (others)	*aclA*	citrate synthase	Reductive TCA cycle
CO2 fixation (others)	*hbsC*	Hydroxybutyryl-CoA Synthase	4-hydroxybutyrate cycles (and Hydroxypropionate-Hydroxybutyrate Cycle)
CO2 fixation (others)	*mcmA*	Methylmalonyl-CoA Mutase	Hydroxypropionate-Hydroxybutyrate Cycle
CO2 fixation (others)	*AcsB*	acetyl-CoA synthase	Wood−Ljungdahl pathway
Ferredoxins	*petF*	Ferredoxin	
Ferredoxins	*petH*	Ferredoxin--NADP reductase	HiPIPs
Ferredoxins	*fdxA*	Ferredoxin	
Ferredoxins	*fdxH*	Ferredoxin	
Ferredoxins	*fdxN*	Ferredoxin-like protein in nif region	HiPIPs
Ferredoxins	*fldA*	Flavodoxin	
Ferredoxins	*hyaB*	Hydrogenase-1 large chain	

Ferredoxins	*narH*	Respiratory nitrate reductase 1 beta chain	
Ferredoxins	*petJ*	Cytochrome c6	HiPIPs
Pigments	*chlL*	Light-independent protochlorophyllide reductase iron-sulfur ATP-binding protein	Chlorophyll
Pigments	*bchX*	Chlorophyllide reductase iron protein subunit	Bacteriochlorophyll
Pigments	*bchY*	Chlorophyllide reductase	Bacteriochlorophyll
Pigments	*bchZ*	Chlorophyllide reductase	Bacteriochlorophyll
Pigments	*bchD*	Mg-protoporphyrin IX chelatase	Bacteriochlorophyll
Pigments	*bchN*	Light-independent protochlorophyllide reductase subunit N	Bacteriochlorophyll
Pigments	*bchF*	2-vinyl bacteriochlorophyllide hydratase	Bacteriochlorophyll
Pigments	*crtB*	Phytoene synthase	Carotenoids
Pigments	*crtL*	Lycopene beta cyclase	Carotenoids
Pigments	*crtE*	Geranylgeranyl pyrophosphate synthase	Carotenoids
Photolyase	*phrA*	CPD Photolyases	
Photolyase	*phrB*	(6-4) Photolyase	
Extracellular polymeric substance (EPS)	*epsA*	EPS I polysaccharide export outer membrane protein EpsA	
Extracellular polymeric substance (EPS)	*epsB*	Putative tyrosine-protein kinase EpsB	
Extracellular polymeric substance (EPS)	*epsE*	EPS I polysaccharide export inner membrane protein EpsE	
Extracellular polymeric substance (EPS)	*epsR*	Negative regulator of exopolysaccharide production protein	
Chemotaxis	*fliC*	Flagellin	
Chemotaxis	*matA*	HTH-type transcriptional regulator EcpR	Pilus
Chemotaxis	*acpA*	Common pilus major fimbrillin subunit EcpA	Pilus
Chemotaxis	*cheA*	Chemotaxis protein CheA	Chemotaxis
Chemotaxis	*tsr*	Methyl-accepting chemotaxis protein I	Chemotaxis
Chemotaxis	*pilA*	Type IV major pilin protein PilA	Pilus
Chemotaxis	*pilB*	Type IV pilus assembly ATPase PilB	Pilus
Chemotaxis	*pilH*	Protein PilH	Pilus
Chemotaxis	*flp*	Flp/Fap pilin component	Pilus
Chemotaxis	*pulO*	Prepilin leader peptidase/N-methyltransferase	Pilus
Chemotaxis	*mcp*	Methyl-accepting chemotaxis protein	Chemotaxis
Branched-chain alpha-keto acid dehydrogenase complex (BCKDC)	*bkdA*	E1α subunit of Branched-Chain α-Keto Acid Decarboxylase	
Branched-chain alpha-keto acid dehydrogenase complex (BCKDC)	*bkdB*	E1β subunit of Branched-Chain α-Keto Acid Decarboxylase	
Branched-chain alpha-keto acid dehydrogenase complex (BCKDC)	*bkdC*	Dihydrolipoyl Transacylase	
Branched-chain alpha-keto acid dehydrogenase complex (BCKDC)	*bkdR*	Bkd operon transcriptional regulator	

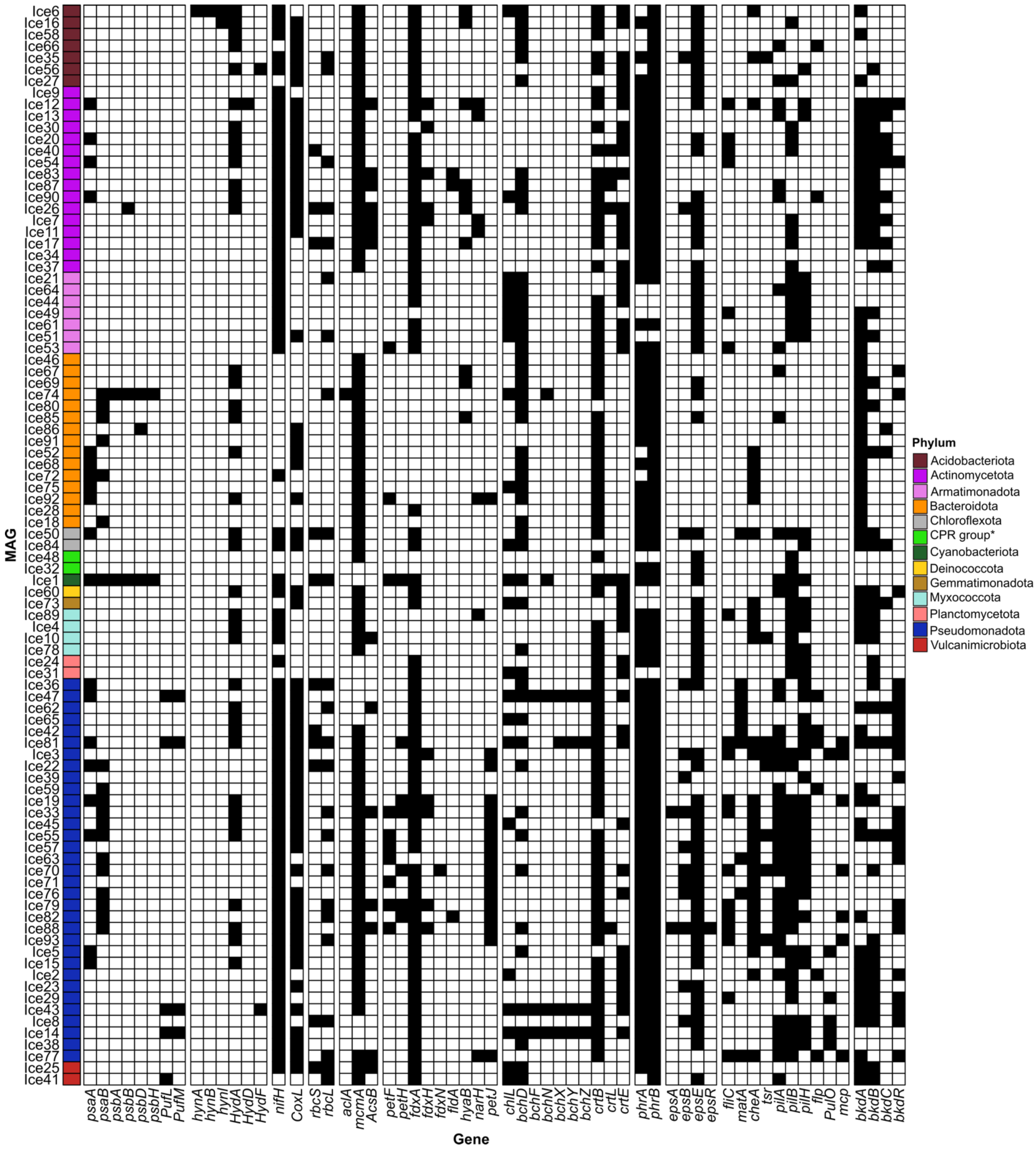

Supplementary Results Figure 1. Genes present (black) and absent (white) in metagenome-assembled genomes (MAGs). More information on the displayed genes can be found in Table S4. Genes were selected to have an overview of potential energy metabolisms and adaptations to the glacial environment. Photosystem I: *psaA* and *psaB*. Photosystem II: *psbA*, *psbB*, *psbD*, *psbH*, *PufL* and *PufM*. Hydrogenase: *hynA*, *hynB*, *hynl*, *HydA*, *HydD* and *HydF*. Nitrogenase: *nifH*. Carbon monoxide dehydrogenase: *CoxL*. CO_2 fixation (Calvin cycle): *rbcS* and *rbcL*. CO_2 fixation (others): *aclA*, *mcmA* and *AcsB*. Ferredoxins: *petF*, *petH*, *fdxA*, *fdxH*, *fdxN*, *fldA*, *hyaB*, *narH* and *petJ*. Pigments: *chlL*, *bchD*, *bchF*, *bchN*, *bchX*, *bchY*, *bchZ*, *crtB*, *crtL* and *crtE*. Photolyase: *phrA* and *phrB*. EPS: *epsA*, *epsB*, *epsE* and *epsR*. Movement and chemotaxis: *fliC*, *matA*, *cheA*, *tsr*, *pilA*, *pilB*, *pilH*, *flp*, *PulO* and *mcp*. Branched-chain alpha-keto acid dehydrogenase complex (BCKDC): *bkdA*, *bkdB*, *bkdC* and *bkdR*. * Not phylum-level.

1. Collins, T. & Margesin, R. Psychrophilic lifestyles: mechanisms of adaptation and biotechnological tools. *Appl. Microbiol. Biotechnol.* **103**, 2857–2871 (2019).
2. Bay, S. K. *et al.* Trace gas oxidizers are widespread and active members of soil microbial communities. *Nat. Microbiol.* **6**, 246–256 (2021).
3. The UniProt Consortium *et al.* UniProt: the Universal Protein Knowledgebase in 2023. *Nucleic Acids Res.* **51**, D523–D531 (2023).
4. Buchfink, B., Xie, C. & Huson, D. H. Fast and sensitive protein alignment using DIAMOND. *Nat. Methods* **12**, 59–60 (2015).
5. Komárek, J., Kaštovský, J., Mareš, J. & Johansen, J. R. Taxonomic classification of cyanoprokaryotes (cyanobacterial genera) 2014, using a polyphasic approach. *Preslia* **86**, 295–335 (2014).
6. Kasalický, V. *et al.* Aerobic Anoxygenic Photosynthesis Is Commonly Present within the Genus Limnohabitans. *Appl. Environ. Microbiol.* **84**, e02116-17 (2018).
7. Mujakić, I. *et al.* Common Presence of Phototrophic *Gemmatimonadota* in Temperate Freshwater Lakes. *mSystems* **6**, e01241-20 (2021).
8. Saggu, S. K., Nath, A. & Kumar, S. Myxobacteria: biology and bioactive secondary metabolites. *Res. Microbiol.* **174**, 104079 (2023).
9. Shivaji, S. & Prakash, J. S. S. How do bacteria sense and respond to low temperature? *Arch. Microbiol.* **192**, 85–95 (2010).
10. Essen, L. O. & Klar, T. Light-driven DNA repair by photolyases. *Cell. Mol. Life Sci.* **63**, 1266–1277 (2006).
11. Marizcurrena, J. J. *et al.* Searching for novel photolyases in UVC-resistant Antarctic bacteria. *Extremophiles* **21**, 409–418 (2017).
12. Halbach, L. *et al.* Pigment signatures of algal communities and their implications for glacier surface darkening. *Sci. Rep.* **12**, 17643 (2022).
13. Hassan, N. *et al.* Glaciochemistry and Pigment Producing Ability of Bacteria from the Roof of the World, the Glaciers of Karakoram, Pakistan. *Geomicrobiol. J.* **40**, 143–151 (2023).
14. Tsuda, K., Nagano, H., Ando, A., Shima, J. & Ogawa, J. Modulation of fatty acid composition and growth in Sporosarcina species in response to temperatures and exogenous branched-chain amino acids. *Appl. Microbiol. Biotechnol.* **101**, 5071–5080 (2017).
15. Xia, Y., Kong, Y., Thomsen, T. R. & Halkjær Nielsen, P. Identification and Ecophysiological Characterization of Epiphytic Protein-Hydrolyzing *Saprospiraceae* (“ *Candidatus* Epiflobacter” spp.) in Activated Sludge. *Appl. Environ. Microbiol.* **74**, 2229–2238 (2008).
